# Quantification of uncertainties in reference and relative dose measurements, dose calculations, and patient setup in modern external beam radiotherapy

**DOI:** 10.1007/s12194-024-00856-0

**Published:** 2024-11-14

**Authors:** Naoki Kinoshita, Morihito Shimizu, Kana Motegi, Yusuke Tsuruta, Toru Takakura, Hiroshi Oguchi, Chie Kurokawa

**Affiliations:** 1 Department of Radiology, Faculty of Medical Sciences, University of Fukui, 23-3, Matsuoka-Shimoaituki, Eiheiji, Yoshida, Fukui 910-1193 Japan; 2 National Metrology Institute of Japan, AIST, Tsukuba, Japan; 3 Section of Radiation Safety and Quality Assurance, National Cancer Center Hospital East, Kashiwa, Japan; 4 Division of Clinical Radiology Service, Kyoto University Hospital, Kyoto, Japan; 5 Seikeikai Medical College, Sakai, Japan; 6 Radiological Technology Department, Iida Municipal Hospital, Iida, Japan; 7 Department of Radiological Technology, Faculty of Health Science, Juntendo University, Tokyo, Japan

**Keywords:** External photon beam radiotherapy, Uncertainty, Reference dosimetry, Relative dosimetry, Dose calculations, Patient setup

## Abstract

Uncertainties in the steps of external beam radiotherapy (EBRT) affect patient outcomes. However, few studies have investigated major contributors to these uncertainties. This study investigated factors contributing to reducing uncertainty in delivering a dose to a target volume. The EBRT process was classified into four steps: reference dosimetry, relative dosimetry [percentage depth doses (PDDs) and off-center ratios (OCRs)], dose calculations (PDDs and OCRs in a virtual water phantom), and patient setup using an image-guided radiation therapy system. We evaluated the uncertainties for these steps in conventionally fractionated EBRT for intracranial disease using 4-, 6-, and 10-MV flattened photon beams generated from clinical linear accelerators following the Guide to the Expression of Uncertainty in Measurement and an uncertainty evaluation method with uncorrected deflection. The following were the major contributors to these uncertainties: beam quality conversion factors for reference dosimetry; charge measurements, chamber depth, source-to-surface distance, water evaporation, and field size for relative dosimetry; dose calculation accuracy for the dose calculations; image registration, radiation-imaging isocenter coincidence, variation in radiation isocenter due to gantry and couch rotation, and intrafractional motion for the patient setup. Among the four steps, the relative dosimetry and dose calculation (namely, both penumbral OCRs) steps involved an uncertainty of more than 5% with a coverage factor of 1. In the EBRT process evaluated herein, the uncertainties in the relative dosimetry and dose calculations must be reduced.

**Supplementary Information:**

The online version contains supplementary material available at 10.1007/s12194-024-00856-0.

## Introduction

Cancer is the second most common cause of death worldwide, accounting for an estimated 9.6 million deaths in 2018 [1]. According to the World Cancer Report 2020 [2], the number of new global cancer cases is expected to exceed 27 million annually by 2040.

Radiotherapy can be an effective cancer treatment; however, the outcome of radiotherapy depends on the accuracy of delivering the prescribed doses to patients. The importance of this accuracy has been recognized since the publication of Report 24 of the International Commission on Radiation Units and Measurements (ICRU) [3]. Recommendations regarding the accuracy of radiotherapy have been established [3–5].

Acceptable accuracy levels are between 3.5 and 5% [coverage factor (*k*) of 1] [4–9]. However, the achievable accuracy is significantly related to uncertainties in radiotherapy performed in clinics. In the early 2000s, uncertainties in external beam radiotherapy (EBRT) with high-energy photon beams were estimated to be 5–6% [9, 10].

EBRT has significantly advanced since the early 2000s. As such, the uncertainty in each step of the EBRT process may vary annually. For example, accurate dosimetry protocols [11, 12], new ionization chamber models, and new recommendations [13] have been developed for reference dosimetry. A wide range of dosimetry equipment (detectors and three-dimensional water phantom systems) is available for relative dosimetry. New dose calculation algorithms, such as the Acuros XB [14] (AXB) (Varian Medical Systems, USA), have recently been implemented in radiotherapy planning. The use of image-guided radiotherapy (IGRT) has increased significantly [15–19].

Recently, international organizations have reported uncertainties in modern EBRT [20, 21]. Furthermore, an organization concluded that each clinic should estimate the uncertainties in its treatment procedures [21]. However, to the best of our knowledge, only few clinical studies have reported practical uncertainties. We evaluated the uncertainties in the conventionally fractionated EBRT process for intracranial disease using 4-, 6-, and 10-MV flattened photon beams generated by a clinical linear accelerator (Linac). This study investigated the major contributions of these uncertainties to improving the accuracy of the dose delivered during radiotherapy.

## Materials and methods

### Uncertainty evaluations

Based on the ICRP Publication 86 [9], the EBRT process was classified into four steps: reference dosimetry, relative dosimetry, dose calculations using a treatment planning system (TPS), and patient setup during dose delivery. In each step, two types of uncertainties were estimated.

- Realistic estimates (i.e., practical uncertainties): These were estimated from our previous work—experimental data [22], quality assurance (QA) results [23]—patient data [24], and values stated in the product datasheets, publications [25–29], and calibration certificates used in our clinics.
- Conservative estimates: These values were estimated from the tolerances and data reported in previous publications [22, 25, 27–38].

The shapes and widths of the probability density functions (PDFs) of the uncertainty components were estimated as follows:

- The variation limits $\pm a$ (e.g., tolerances stated in publications) of the components were known, but the shapes of their PDFs were unknown. The shapes of these PDFs were assumed to be uniform within $\pm a$.
- When the standard deviations, σ, of the components were derived from the experimental data on the QA and uncertainties obtained from the previous publications, their PDFs were presumed to be normal PDFs with standard deviations of σ.

The uncertainties in the steps were derived from the PDFs using the Guide to the Expression of Uncertainty in Measurement [39] and an uncertainty evaluation method with uncorrected deflection [40].

### Uncertainty components and their PDFs

#### Reference dosimetry

The uncertainties in the measurements of the absorbed dose to water under the reference conditions [a field size of 10 × 10 cm^2^ at a source-chamber distance (SCD) of 100 cm, chamber depth of 10 cm, and relative humidity range of 20–80%] were estimated. These measurements were performed using a TrueBeam linac (Varian Medical Systems, USA), PTW 30013 chamber (PTW-Freiburg, Germany), WP1D water phantom (IBA Dosimetry, Germany), and RAMTEC Duo electrometer (Toyo Medic, Japan), following the Standard Dosimetry of Absorbed Dose to Water in External Beam Radiotherapy (Standard Dosimetry 12) [27] and Japan Society of Medical Physics (JSMP) guidelines on electrometers used for radiotherapy dosimeters (JSMP Electrometer Guidelines 17) [25].

Table 1 presents the uncertainty components associated with the absorbed dose to water considered in this study. Additional information about Table 1 is provided as follows:

- Reference conditions
  - SCD setting: This setting was achieved using two laser units with a localization accuracy of ± 1 mm on the lateral walls of the linac treatment room. The PDF was assumed to be uniform within ± 1 mm.
  - Field size: The individual jaw positions on the linac agreed with the desired setting (i.e., X-jaw = 10 cm and Y-jaw = 10 cm at SCD = 100 cm) within ± 0.5 mm. The PDF was presumed to be uniform within ± 1 mm (the sum of the variations in the X- and Y-jaw positions).
- Charge reading
  - Electrometer: The uncertainty of the electrometer used in this study could not be evaluated. Therefore, we used an uncertainty of 0.35% [25] in the charge measurement performed using a reference-class electrometer with capacitor negative feedback in the operational amplifier.
  - ${M}_{raw}$ relative to 100 MU: This PDF was determined to be a normal PDF with the standard deviation of the mean chamber reading (${M}_{raw}$) relative to 100 monitor units (MU).
- Correction for influence quantities
  - Instrumental errors in the barometer and thermometer: These errors would not exceed the verification tolerances [26] of ± 0.07 kPa and ± 0.5 ℃ for pressure and temperature measurements, respectively. These tolerances were assumed to be the variation limits of the uniform PDFs for pressure and temperature measurements.
  - $\frac{{M}_{+}}{{M}_{-}}$: This PDF was derived from the root sum square of the standard deviation of the mean chamber readings, ${\text{M}}_{+}$ and ${\text{M}}_{-}$, obtained at polarizing voltages of $-$ 300 and $+$ 300 V, respectively.
  - $\frac{{M}_{1}}{{M}_{2}}$: This PDF was derived from the root sum square of the standard deviation of the mean-collected charges, ${\text{M}}_{1}$ and ${\text{M}}_{2}$, obtained at polarizing voltages of $+$ 300 and $+$ 100 V, respectively.
- Determination of beam quality conversion factor (${k}_{Q,{Q}_{0}}$)
  - ${M}_{raw}$ relative to 100 MU at depths of 10 and 20 cm: These PDFs were determined in the same manner as ${M}_{raw}$ relative to 100 MU (as explained above).
  - Difference in ${s}_{w,air}$: As observed in the literatures [28, 29], the differences between the water-to-air stopping-power ratios (${s}_{w,air}$) resulting from the updated values (described in ICRU Report 90 [13]) for the mean excitation energy of water (*I*_water_) and from the present values (described in ICRU Report 37 [41]) for *I*_water_ are − 0.6% and − 0.4% for ^60^Co γ-rays and MV photons, respectively. The ratio of the updated ${s}_{w,air}$ for ^60^Co γ-rays to that for the MV photons would be 0.2% lower than the ratio of the present ${s}_{w,air}$ for ^60^Co γ-rays to that for the MV photons.

**Table 1:** Uncertainty components associated with absorbed dose to water for the 4-, 6-, and 10-MV photons and their PDFs

Component of uncertainty	PDFs of realistic estimate		PDFs of conservative estimate	
	Shape	Variation limit/standard deviation/deflection	Shape	Variation limit/ standard deviation/ deflection
1: Reference conditions				
1-1: SCD setting	Uniform	± 1 mm^a^	Uniform	± 2 mm^k^
1-2: Chamber setting	–		Normal	± 0.5 mm^l^
1-2-1: Setting origin in phantom	Uniform	± 0.4 mm^b^	–	–
1-2-2: Position accuracy	Uniform	± 0.2 mm^c^	–	–
1-2-3: Position reproducibility	Uniform	± 0.1 mm^c^	–	–
1-3: Field-size setting	Uniform	± 1 mm^a^	Uniform	± 2 mm^m^
2: Charge measurement			Normal	± 0.6%^g^
2-1: Electrometer	Normal	± 0.35%^d^	–	–
2-2: ${M}_{raw}$ relative to 100 MU	Normal Normal Normal	± 0.01%^a^ for 4 MV ± 0.01%^a^ for 6 MV ± 0.005%^a^ for 10 MV	– – –	– – –
3: Long-term stability of ion chamber	Uniform	± 0.2%^e^	Normal	± 0.3%^g^
4: Correction for influence quantities				
4-1: Pressure and temperature				
4-1-1: Instrument error in thermometer	Uniform	± 0.5 ℃^f^	Normal	± 0.3 ℃^n^
4-1-2: Instrument error in barometer	Uniform	± 0.07 kPa^f^	Normal	± 0.1 kPa^n^
4-2: Polarity effect			Normal	± 0.05%^l^
4-2-1:$\frac{{M}_{+}}{{M}_{-}}$	Normal Normal Normal	± 0.02%^a^ for 4 MV ± 0.01%^a^ for 6 MV ± 0.005%^a^ for 10 MV	– – –	– – –
4-3: Ion Recombination			Normal	± 0.10%^l^
4-3-1:$\frac{{M}_{1}}{{M}_{2}}$	Normal Normal Normal	± 0.02%^a^ for 4 MV ± 0.01%^a^ for 6 MV ± 0.008%^a^ for 10 MV	– – –	– – –
4-4: Humidity	Normal	± 0.15%^g^	Normal	± 0.15%^g^
5: Calibration of dosimeter				
5-1: Ion chamber	Normal	± 1.0%^h^	Normal	± 0.49%^o^
5-2: Electrometer	Normal	± 0.15%^h^	Normal	± 0.09%^o^
6: Determination of ${k}_{Q,{Q}_{0}}$				
6-1: *TPR*_*20,10*_				
6-1-1: Chamber position at 10-cm depth	Normal	± 0.26 mm^i^	Normal	± 0.5 mm^l^
6-1-2: ${M}_{raw}$ relative to 100 MU at 10-cm depth	Normal Normal Normal	± 0.01%^a^ for 4 MV ± 0.01%^a^ for 6 MV ± 0.005%^a^ for 10 MV	Normal	± 0.05%^l^
6-1-3: Chamber position at 20-cm depth	Normal	± 0.26 mm^i^	Normal	± 0.5 mm^l^
6-1-4: ${M}_{raw}$ relative to 100 MU at 20-cm depth	Normal Normal Normal	± 0.01%^a^ for 4 MV ± 0.007%^a^ for 6 MV ± 0.005%^a^ for 10 MV	Normal	± 0.05%^l^
6-2:${k}_{Q,{Q}_{0}}$	Normal	± 1.0%^g^	Normal	± 1.0%^g^
6-3: Difference in ${s}_{w,air}$	Deflection	0.2%^j^	Deflection	0.2%^j^

The variation limits of uniform PDFs and standard deviations of normal PDFs, except for those related to humidity, calibration of dosimeter, and ${k}_{Q,{Q}_{0}}$, can vary among clinics

^a^QA results derived from our previous work

^b^Experimental data derived from our previous study [22]: variation in setting origin for phantoms among users

^c^Values stated in a product datasheet used here

^d^A value stated in Ref. [25]: the uncertainty of reference-class electrometers which rely on charge storage capacitor

^e^Experimental data derived from our previous work: this value was obtained from chamber-calibration data in a dosimetry calibration laboratory

^f^Values stated in Ref. [26]: verification tolerances for barometers and thermometers

^g^Uncertainties (*k* = 1) stated in Ref [27]

^h^Uncertainties (*k* = 2) stated in calibration certificates used here

^i^Derived from combining three components: components 1-2-1, 1-2-2, and 1-2-3 in the realistic estimate of Table 1

^j^A value stated in Refs. [28, 29]: the difference in ${s}_{w,air}$ resulting from the ICRU 90 *I*_water_ and from the ICRU 37 *I*_water_

^k^A tolerance stated in Ref. [30]: laser localization

^l^Uncertainties (*k* = 1) presented in Ref [31]

^m^A tolerance stated in Ref. [30]: jaw position indicators

^n^Uncertainties (*k* = 1) presented in Ref. [32]. ^o^Uncertainties (*k* = 1) presented in Ref. [25]

#### Relative dosimetry

The following uncertainties were estimated under a field size of 10 × 10 cm^2^ at a 100-cm source-to-surface distance (SSD) established using a TrueBeam linac (Varian Medical Systems, USA), MP3-M three-dimensional water tank, and PTW 31021 chamber (PTW-Freiburg, Germany):

- Percentage depth doses (PDDs) at depths of 0.6, 0.7, and 1.2 cm (buildup [42] for 4, 6, and 10-MV photons, respectively) and 10 cm (inner [42]) along the central beam axis.
- Off-center ratios (OCRs) at 4 cm (inner [42]) and 5.5 cm (penumbra [42]) off axis from the central beam axis at a depth of 10 cm.

Tables 2 and 3 present the uncertainty components of the PDDs and OCRs estimated in this study, respectively. The PDFs for the field size and instrumental errors in the barometer and thermometer were estimated in the same manner as in reference dosimetry (see Sect. 2.2.1). Additional information on Tables 2 and 3 is provided as follows:

- SSD setting: This setting was achieved using two laser units with a localization accuracy of ± 1 mm on the lateral walls of the linac treatment room. The PDF was assumed to be uniform within ± 1 mm.
- Water evaporation
  - Water level adjustment among users: The water level was adjusted by adding water whenever a 1-mm change in water level occurred. The uncertainty in this adjustment was estimated.
  - Mean displacement after water level adjustment: This displacement would be 0.5 mm because the change in water level would be $\le 1$ mm.
  - Variation in water level: Considering the change in the water level, this variation would be ± 0.5 mm from the mean displacement.
- Charge measurement
  - Electrometer readings in the denominator: The sources associated with this uncertainty were uncertainty components [25] for electrometers with a resistor in the operational amplifier, except for long-term stability, change in response due to temperature, mains voltage fluctuation (static), and cable effect (see component 2–1 in Tables 2 and 3). The uncertainties in the long-term stability, change in response, mains voltage fluctuation (static), and cable effects were canceled in the numerator and denominator of the PDDs and OCRs.
  - Electrometer readings in the numerator: The repeatability and zero drift can vary during the PDD and OCR measurements. These components were associated with this uncertainty.
- Temperature–pressure corrections in the denominator and numerator: Temperature and pressure varied during the PDD and OCR measurements. These components were associated with these uncertainties.

**Table 2:** Uncertainty components associated with PDDs for the 4-, 6-, and 10-MV photons and their PDFs

Component of uncertainty	PDFs of realistic estimate		PDFs of conservative estimate	
	Shape	Variation limit/standard deviation/deflection	Shape	Variation limit/standard deviation/seflection
1: Measurement conditions				
1-1: SSD setting	Uniform	± 1 mm^a^	Uniform	± 2 mm^h^
1-2: Setting chamber at depth of maximum dose			Normal	± 0.5 mm^i^
1-2-1: Setting origin in phantom	Uniform	± 0.4 mm^b^	–	–
1-2-2: Reproducibility of movement distance	Uniform	± 0.1 mm^c^	–	–
1-3: Setting chamber at 0.6 cm/0.7 cm/1.2 cm/10 cm depth				
1-3-1: Accuracy of movement distance	–	–	Uniform	± 0.1 mm^j^
1-3-2: Reproducibility of movement distance	Uniform	± 0.1 mm^c^	–	
1-4: Field-size setting	Uniform	± 1 mm^a^	Uniform	± 2 mm^k^
1-5: Water evaporation				
1-5-1: Water level adjustment among users	Uniform	± 0.4 mm^d^	Uniform	± 0.4 mm^d^
1-5-2: Mean displacement after water level adjustment	Deflection	0.5 mm	Deflection	0.5 mm
1-5-3: Variation in water level	Uniform	± 0.5 mm	Uniform	± 0.5 mm
2: Charge measurement				
2-1: Electrometer reading in denominator				
2-1-1: Display resolution^e^	Normal	± 0.058%^f^	Normal	± 0.058%^f^
2-1-2: Repeatability^e^	Normal	± 0.1%^f^	Normal	± 0.1%^f^
2-1-3: Zero drift^e^	Normal	± 0.058%^f^	Normal	± 0.058%^f^
2-1-4: Non-linearity^e^	Normal	± 0.12%^f^	Normal	± 0.12%^f^
2-1-5: Response to pulsed beam from a linac^e^	Normal	± 0.12%^f^	Normal	± 0.12%^f^
2-1-6: Stabilization time^e^	Normal	± 0.12%^f^	Normal	± 0.12%^f^
2-1-7: Mains voltage fluctuation during measurement^e^	Normal	± 0.12%^f^	Normal	± 0.12%^f^
2-1-8: Elapsed timer^e^	Normal	± 0.01%^f^	Normal	± 0.01%^f^
2-2: Electrometer reading in numerator				
2-2-1: Repeatability^e^	Normal	± 0.1%^f^	Normal	± 0.1%^f^
2-2-2: Zero drift^e^	Normal	± 0.058%^f^	Normal	± 0.058%^f^
3: Correction for influence quantities				
3-1: Temperature–pressure correction in denominator				
3-1-1: Instrument error in thermometer	Uniform	± 0.5 ℃^g^	Normal	± 0.3 ℃^l^
3-1-2: Instrument error in barometer	Uniform	± 0.07 kPa^g^	Normal	± 0.1 kPa^l^
3-2: Temperature–pressure correction in numerator				
3-2-1: Instrument error in thermometer	Uniform	± 0.5 ℃^g^	Normal	± 0.3℃^l^
3-2-2: Instrument error in barometer	Uniform	± 0.07 kPa^g^	Normal	± 0.1 kPa^l^

The variation limits of uniform PDFs and deflection can vary among clinics

^a^QA results derived from our previous work

^b^Experimental data derived from our previous study [22]: variation in setting origin for phantoms among users

^c^A value stated in a product datasheet used herein

^d^Experimental data derived from our previous study [22]: variation in water level adjustment among users

^e^The performance characteristics of electrometers, which are explained in Ref [25]

^f^Uncertainties (*k* = 1) presented in Ref [25]

^g^Values stated in Ref. [26]: verification tolerances for barometers and thermometers

^h^A tolerance stated in Ref. [30]: laser localization

^i^An uncertainty presented in Ref. [31]. ^j^A tolerance stated in Ref. [35]: accuracy of movement distance

^k^A tolerance stated in Ref. [30]: jaw position indicators. ^l^Uncertainties (*k* = 1) presented in Ref. [32]

**Table 3:** Uncertainty components associated with OCRs for the 4-, 6-, and 10-MV photons and their PDFs. The variation limits of uniform PDFs and deflection can vary among clinics

Component of uncertainty	PDFs of realistic estimate		PDFs of conservative estimate	
	Shape	Variation limit/standard deviation/deflection	Shape	Variation limit/standard deviation/deflection
1: Measurement conditions				
1-1: SSD setting	Uniform	± 1 mm^a^	Uniform	± 2 mm^h^
1-2: Chamber setting on beam axis at 10 cm depth			Normal	± 0.5 mm^i^
1-2-1: Setting origin in phantom	Uniform	± 0.4 mm^b^	–	–
1-2-2: Accuracy of movement distance	Uniform	± 0.1 mm^c^	–	–
1-3: Chamber setting at 4 cm/5.5 cm off axis				
1-3-1: Accuracy of movement distance	Uniform	± 0.1 mm^c^	Uniform	± 0.1 mm^j^
1-4: Field-size setting				
1-4-1: Chamber reading on beam axis at 10 cm depth	Uniform	± 1 mm^a^	Uniform	± 2 mm^k^
1-4-2: Chamber reading at 4 cm/5.5 cm off axis	Uniform	± 1 mm^a^	Uniform	± 2 mm^k^
1-5: Water evaporation				
1-5-1: Water level adjustment among users	Uniform	± 0.4 mm^d^	Uniform	± 0.4 mm^d^
1-5-2: Mean displacement from water level adjustment	Deflection	0.5 mm	Deflection	0.5 mm
1-5-3: Variation in water level	Uniform	± 0.5 mm	Uniform	± 0.5 mm
2: Charge measurement				
2-1: Electrometer reading in denominator				
2-1-1: Display resolution^e^	Normal	± 0.058%^f^	Normal	± 0.058%^f^
2-1-2: Repeatability^e^	Normal	± 0.1%^f^	Normal	± 0.1%^f^
2-1-3: Zero drift^e^	Normal	± 0.058%^f^	Normal	± 0.058%^f^
2-1-4: Non-linearity^e^	Normal	± 0.12%^f^	Normal	± 0.12%^f^
2-1-5: Response to pulsed beam from a linac^e^	Normal	± 0.12%^f^	Normal	± 0.12%^f^
2-1-6: Stabilization time^e^	Normal	± 0.12%^f^	Normal	± 0.12%^f^
2-1-7: Mains voltage fluctuation during measurement^e^	Normal	± 0.12%^f^	Normal	± 0.12%^f^
2-1-8: Elapsed timer^e^	Normal	± 0.01%^f^	Normal	± 0.01%^f^
2-2: Electrometer reading in numerator				
2-2-1: Repeatability^e^	Normal	± 0.1%^f^	Normal	± 0.1%^f^
2-2-2: Zero drift^e^	Normal	± 0.058%^f^	Normal	± 0.058%^f^
3: Correction for influence quantities				
3-1: Temperature–pressure correction in denominator				
3-1-1: Instrument error in thermometer	Uniform	± 0.5 ℃^g^	Normal	± 0.3 ℃^l^
3-1-2: Instrument error in barometer	Uniform	± 0.07 kPa^g^	Normal	± 0.1 kPa^l^
3-2: Temperature–pressure correction in numerator				
3-2-1: Instrument error in thermometer	Uniform	± 0.5 ℃^g^	Normal	± 0.3 ℃^l^
3-2-2: Instrument error in barometer	Uniform	± 0.07 kPa^g^	Normal	± 0.1 kPa^l^

^a^QA results derived from our previous work

^b^Experimental data derived from our previous study [22]: variation in setting origin for phantoms among users

^c^A value stated in a product datasheet used herein

^d^Experimental data derived from our previous study [22]: variation in water level adjustment among users

^e^Performance characteristics of electrometers, which are explained in Ref. [25]

^f^Uncertainties (*k* = 1) presented in Ref. [25]

^g^Values stated in Ref. [26]: verification tolerances for barometers and thermometers

^h^A tolerance stated in Ref. [30]: laser localization

^i^An uncertainty presented in Ref. [31]

^j^A tolerance stated in Ref. [35]: accuracy of movement distance

^k^A tolerance stated in Ref. [30]: jaw position indicators

^l^Uncertainties (*k* = 1) presented in Ref. [32]

#### Dose calculations in TPS

The following uncertainties were estimated under a 10 × 10 cm^2^ field established by the X- and Y-jaws on a TrueBeam linac at an SSD of 100 cm.

- PDDs in buildup and inner [42] regions of the central beam axis.
- OCRs in inner and penumbra [42] regions of beam profile at a depth of 10 cm.

Modern radiotherapy involves a complex treatment delivery process [21]. Consequently, the uncertainty in the calculation of the dose distribution delivered to a patient cannot easily be evaluated. Therefore, the uncertainties in the calculations of the PDDs and OCRs were estimated using homogeneous and simple geometries.

Tables 4 and 5 present the uncertainty components of the calculations performed using AXB [14] with a dose-to-medium dose-reporting mode equipped with Eclipse ver. 15 (Varian Medical Systems, USA), which were considered here. Additional information related to these components is provided as follows:

- CT number: To obtain the realistic estimates presented in Tables 4 and 5, a daily QA phantom (Siemens, Germany) filled with water was scanned using a SOMATOM Definition AS instrument (Siemens, Germany). Five regions of interest (ROIs) were set at the center, top, bottom, right, and left of each image acquired during the daily QA tests. The average CT value was calculated using the CT numbers of these five ROIs. The variation in the average CT numbers was approximately ± 0.5 Hounsfield units (HU). The PDF for the average CT number was presumed to be uniform within ± 0.5 HU.
- PDD in the buildup/inner region and OCR in the inner/penumbra region: The dose differences, ${\delta }_{calc, meas} \left(\%\right)$, between the calculated doses (depth and off-axis doses), $D_{calc}$ and measured doses (depth and off-axis doses), ${D}_{meas}$, were estimated as follows:
  $$\delta_{calc,meas} (\% ) = \frac{{\left( {D_{calc} - D{}_{meas}} \right)}}{{D_{meas} }} \times 100$$

PDFs for the PDDs in the buildup/inner regions and OCRs in the inner/penumbra regions were estimated from the maximum ${\delta }_{calc, meas}$. Measurements and calculations of ${D}_{meas}$ and ${D}_{calc}$ were performed as follows:

Measurements: The measurements were performed using a CC04 chamber and SMARTSCAN phantom (IBA Dosimetry, Germany) at a gantry angle of 0°and an SSD of 100 cm. The CC04 was positioned parallel to the beam axis in the phantom.

Calculations: The calculations were performed using a virtual water phantom with a gantry angle of 0°and SSD of 100 cm. The CT value and material of this phantom in Eclipse were 0 HU and water, respectively.

- Beam attenuation by the treatment couch: The percentage difference (%Dif) between the attenuation rates determined by calculations and measurements was evaluated as follows:
  $$\% {\text{Dif}} = \frac{{\left( {A_{c} - A_{m} } \right)}}{{A_{m} }} \times 100 \left( \% \right)$$

where ${A}_{m}$ is the attenuation rate determined by measurements, and ${A}_{c}$ is the attenuation rate determined by calculations. To obtain the realistic estimates presented in Tables 4 and 5, the PDFs for beam attenuation by the thin portion (generally used for head and neck cancer) of the Varian Exact IGRT couch were estimated from the maximum %Dif. ${A}_{m}$ and ${A}_{c}$ were determined as follows:

${A}_{m}$: A 0.6 cm^3^ ionization chamber (PTW-Freiburg, Germany) was inserted into the center of a circular phantom with a radius of 10 cm (Taisei Medical, Inc., Japan) on the thin portion of the couch. The field size was 10 × 10 cm^2^ at an SCD of 100 cm. The gantry was rotated at intervals of 10°, with incident beam angles between 90° and 180°. A total of 100 MU of radiation was applied at each angle. ${A}_{m}$ was calculated as follows:

$$A_{m} \left( \% \right) \, = \left( {1 - \frac{{M_{\theta } }}{{M_{0} }}} \right) \times {1}00$$

where θ represents the gantry angle, and *M*_θ_ and *M*_*0*_ represent chamber readings with gantry angles of θ and 0°, respectively.

${A}_{c}$: A virtual circular phantom was created using Eclipse. The CT value and material of the phantom were 0 HU and water, respectively. Eclipse has an inherent virtual phantom of a treatment couch consisting of a couch surface and couch interior. The assigned CT values for the couch surface and interior were $-$ 300 and $-$ 1000 HU, respectively. No materials were assigned to these structures. ${A}_{c}$ was calculated under the same conditions as the measurements:

$$A_{c} \left( \% \right) \, = \left( {1 - \frac{{C_{\theta } }}{{C_{0} }}} \right) \times {1}00$$

where θ represents the gantry angle, and *C*_θ_ and *C*_*0*_ represent the calculated values with gantry angles of θ and 0°, respectively.

**Table 4:** Uncertainty components associated with the calculations of the PDDs for the 4-, 6-, and 10-MV photons in the buildup and inner regions on the central beam axis and their PDFs

Component of uncertainty	PDFs of realistic estimate		PDFs of conservative estimate	
	Shape	Variation limit	Shape	Variation limit
1: CT number	Uniform	± 0.5 HU^a^	Uniform	± 5 HU^e^
2: PDD in the buildup/inner region	Uniform	± 1.7%^b^/ ± 1.1%^c^ for 4 MV ± 0.90%^b^/ ± 0.50%^c^ for 6 MV ± 2.4%^b^/ ± 1.2%^c^ for 10 MV	Uniform	± 10%^f^/ ± 2%^g^
3: Beam attenuation by treatment couch	Uniform	± 0.44%^d^ for 4 MV ± 0.32%^d^ for 6 MV ± 0.24%^d^ for 10 MV	Uniform	± 0.6%^h^

These variation limits can vary among clinics

^a^QA results derived from our previous work: CT number for water

^b^Experimental data derived from our previous work: difference between measured and calculated doses in the buildup

^c^Experimental data derived from our previous work: difference between measured and calculated doses in the inner

^d^Experimental data derived from our previous work: difference between attenuation rates determined by calculations and measurements

^e^A tolerance stated in Ref. [33]: CT number for water

^f^A tolerance stated in Ref. [36]: accuracy of photon beam dose calculations in a buildup region

^g^A tolerance stated in Ref. [36]: accuracy of photon beam dose calculations in an inner region

^h^A value stated in Ref. [37]: difference between attenuation rates determined by calculations and measurements

**Table 5:** Uncertainty components associated with the calculations of the OCRs for the 4-, 6-, and 10-MV photons in the inner and penumbra regions of the beam profile at a depth of 10 cm and their PDFs

Component of uncertainty	PDFs of realistic estimate		PDFs of conservative estimate	
	Shape	Variation limit	Shape	Variation limit
1: CT number	Uniform	± 0.5 HU^a^	Uniform	± 5 HU^e^
2: OCR in the inner/penumbra region	Uniform	± 3.4%^b^/ ± 26.4%^c^ for 4 MV ± 3.0%^b^/ ± 26.0%^c^ for 6 MV ± 2.4%^b^/ ± 30.4%^c^ for 10 MV	Uniform	± 3%^f^/ ± 10%^g^
3: Beam attenuation by treatment couch	Uniform	± 0.44%^d^ for 4 MV ± 0.32%^d^ for 6 MV ± 0.24%^d^ for 10 MV	Uniform	± 0.6%^h^

These variation limits can vary among clinics

^a^QA results derived from our previous work: CT number for water

^b^Experimental data derived from our previous work: difference between measured and calculated doses in the inner

^c^Experimental data derived from our previous work: difference between measured and calculated doses in the penumbra

^d^Experimental data derived from our previous work: difference between attenuation rates determined by calculations and measurements

^e^A tolerance stated in Ref. [33]: CT number for water

^f^A tolerance stated in Ref. [36]: accuracy of photon beam dose calculations in an inner region

^g^A tolerance stated in Ref. [36]: accuracy of photon beam dose calculations in a penumbra region

^h^A value stated in Ref. [37]: difference between attenuation rates determined by calculations and measurements

#### Patient setup

The following uncertainties in the conventionally fractionated EBRT for intracranial disease were estimated:

- Uncertainties in patient positioning using an ExacTrac X-ray system (Brainlab, Germany)
- Uncertainties in intrafractional head motion

We assumed that patient immobilization for this radiotherapy was performed using a Type-S IMRT (head only) thermoplastic mask with an AccuForm head cushion (CIVCO, USA).

Tables 6 and 7 present the uncertainty components associated with the patient setup and intrafractional head motion, respectively, which were considered here. The standard deviations and deflection of these PDFs in the realistic estimates were obtained from previous work [23, 24]. Intrafractional head motion was evaluated from the positional differences between the patient positions acquired at before and after treatment using a cone-beam CT [24]. Additional information related to these components is as follows.

- Image registration: An uncertainty arose in auto-image registration when the process was repeated 10 times using the same images acquired with the ExacTrac X-ray system [23].
- Coincidence of imaging and radiation isocenters: This coincidence was determined using the method described in our previous work [23].
- Variation in the radiation isocenter due to gantry rotation: This variation was determined using the Winston–Lutz test [43].
- Mean displacement from the imaging isocenter: The displacement due to intrafractional motion, which was assumed to be a deflection in the estimation of this uncertainty, was assessed using the uncertainty evaluation method [40] with uncorrected deflection.

**Table 6:** Uncertainty components associated with patient positioning and their PDFs

Component of uncertainty	PDFs of realistic estimate		PDFs of conservative estimate	
	Shape	Standard deviation	Shape	Variation limit
1: Image registration	Normal	± 0.22 mm^a^	Uniform	± 2 mm^d^
2: Coincidence of imaging and radiation isocenters	Normal	± 0.30 mm^b^	Uniform	± 2 mm^e^
3: Variation in radiation isocenter due to gantry rotation	Normal	± 0.23 mm^c^	Uniform	± 1 mm^f^
4: Variation in radiation isocenter due to couch rotation	Normal	± 0.22 mm^c^	Uniform	± 1 mm^g^

These standard deviations can vary among clinics

^a^An uncertainty (*k* = 1) presented in Ref. [23]: results of auto-image registration

^b^An uncertainty (*k* = 1) presented in Ref. [23]: coincidence between the radiation and imaging isocenters

^c^Uncertainties (*k* = 1) presented in Ref. [23]: position of the radiation isocenter

^d^A tolerance stated in Ref. [34]: accuracy of image registration

^e^A tolerance stated in Ref. [30]: imaging and treatment coordinate coincidence

^f^A tolerance stated in Ref. [30]: variation in radiation isocenter due to gantry rotation

^g^A tolerance stated in Ref. [30]: variation in radiation isocenter due to couch rotation

**Table 7:** Uncertainty components associated with intrafractional head motion and their PDFs

Component of uncertainty	PDFs of realistic estimate		PDFs of conservative estimate	
	Shape	Deflection/standard deviation	Shape	Variation limit
1: Mean displacement from imaging isocenter	Deflection	1.06 mm^a^	–	–
2: Variation in intrafractional motion	Normal	± 1.21 mm^b^	Uniform	± 3 mm^c^

The standard deviation of the normal PDF and deflection can vary among clinics

^a^Data derived from Ref. [24]: head displacement from imaging isocenter

^b^Data derived from Ref. [24]: a standard deviation of intrafractional head motion

^c^A value stated in Ref. [38]: intrafractional head motion for intracranial radiotherapy with a head mask

### Dosimetric and geometric uncertainties in EBRT process

We evaluated dosimetric and geometric uncertainties in the EBRT process based on the American Association of Physicists in Medicine (AAPM) Report 13 [44]. Dosimetric uncertainties were evaluated by combining uncertainties in the reference dosimetry, relative dosimetry, and dose calculations. Geometric uncertainties were derived by combining the uncertainties in patient positioning and intrafractional head motion.

## Results

### Reference dosimetry

Table 8 presents the uncertainties in the absorbed dose to water for the 4-, 6-, and 10-MV photons. Supplementary Tables 1–6 present these budgets. The combined uncertainties (*k* = 1) were approximately 1.2% and 1.4% for realistic and conservative estimates, respectively. The largest contributor to these uncertainties was ${k}_{Q,{Q}_{0}}$.

**Table 8:** Uncertainties in the absorbed dose to water for the 4-, 6-, and 10-MV photons

	Realistic estimate			Conservative estimate		
	4 MV	6 MV	10 MV	4 MV	6 MV	10 MV
Component of uncertainty	Value (%)	Value (%)	Value (%)	Value (%)	Value (%)	Value (%)
1: Reference conditions						
1-1: SCD setting	0.12	0.12	0.12	0.23	0.23	0.23
1-2: Chamber setting	0.18	0.15	0.13	0.33	0.29	0.24
1-3: Field-size setting	0.11	0.09	0.07	0.23	0.17	0.13
2: Charge measurement	0.35	0.35	0.35	0.6	0.6	0.6
3: Long-term stability of ion chamber	0.12	0.12	0.12	0.3	0.3	0.3
4: Correction for influence quantities						
4-1: Pressure and temperature						
4-1-1: Instrument error in thermometer	0.1	0.1	0.1	0.1	0.1	0.1
4-1-2: Instrument error in barometer	0.04	0.04	0.04	0.1	0.1	0.1
4-2: Polarity effect				0.05	0.05	0.05
4-2-1:$\frac{{M}_{+}}{{M}_{-}}$	0.01	0.006	0.003	–	–	–
4-3: Ion Recombination				0.1	0.1	0.1
4-3-1:$\frac{{M}_{1}}{{M}_{2}}$	0.01	0.005	0.004	–	–	–
4-4: Humidity	0.15	0.15	0.15	0.15	0.15	0.15
5: Calibration of dosimeter						
5-1: Ion chamber	0.5	0.5	0.5	0.49	0.49	0.49
5-2: Electrometer	0.075	0.075	0.075	0.09	0.09	0.09
6: Determination of ${k}_{Q,{Q}_{0}}$						
6-1:${TPR}_{\text{20,10}}$	0.01	0.02	0.03	0.02	0.04	0.06
6-2:${k}_{Q,{Q}_{0}}$	1.0	1.0	1.0	1.0	1.0	1.0
6-3: Difference in ${s}_{w,air}$	0.2	0.2	0.2	0.2	0.2	0.2
Combined standard uncertainty	1.23	1.23	1.22	1.42	1.40	1.39
Expanded uncertainty (*k* = 2)	2.5	2.5	2.5	2.9	2.8	2.8

Values except for expanded uncertainties (*k* = 2) are given as relative standard uncertainties (*k* = 1). These relative uncertainties, except for humidity, calibration of dosimeter, ${k}_{Q,{Q}_{0}}$, and difference in ${s}_{w,air}$, can vary among clinics

### Relative dosimetry

#### PDD

Table 9 presents the uncertainties in the PDDs at depths of 0.6, 0.7, and 1.2 cm for the 4-, 6-, and 10-MV photons, respectively. Supplementary Tables 7–12 present these budgets. The combined uncertainties (*k* = 1) were approximately 0.39–0.42% and 0.47–0.49% for the realistic and conservative estimates, respectively. The major uncertainty components were the charge measurement, SSD, and setting chamber at depths of 0.6, 0.7, and 1.2 cm.

**Table 9:** Uncertainties in the PDD measurements in the buildup (0.6-, 0.7-, and 1.2-cm depths for the 4-, 6-, and 10-MV photons, respectively) and inner (10-cm depth) regions on the central axis of the 4-, 6-, and 10-MV photons

	PDD at 0.6 cm/0.7 cm/1.2 cm depth (Buildup region)						PDD at 10 cm depth (Inner region)					
	Realistic estimate			Conservative estimate			Realistic estimate			Conservative estimate		
	4 MV	6 MV	10 MV	4 MV	6 MV	10 MV	4 MV	6 MV	10 MV	4 MV	6 MV	10 MV
Component of uncertainty	Value (%)	Value (%)	Value (%)	Value (%)	Value (%)	Value (%)	Value (%)	Value (%)	Value (%)	Value (%)	Value (%)	Value (%)
1: Measurement conditions												
1-1: SSD setting	0.12	0.12	0.12	0.23	0.23	0.23	0.12	0.12	0.12	0.23	0.23	0.23
1-2: Setting chamber at depth of maximum dose	0.05	0.02	0.02	0.11	0.03	0.04	0.05	0.02	0.02	0.11	0.03	0.04
1-3: Setting chamber at 0.6 cm/0.7 cm/1.2 cm/10 cm depth	0.16	0.19	0.10	0.16	0.19	0.10	0.04	0.03	0.03	0.04	0.03	0.03
1-4: Field-size setting	0.04	0.04	0.04	0.09	0.09	0.09	0.07	0.07	0.06	0.14	0.14	0.12
1-5: Water evaporation	0.10	0.13	0.13	0.10	0.13	0.13	0.25	0.24	0.18	0.25	0.24	0.18
2: Charge measurement												
2-1: Electrometer reading in denominator	0.27	0.27	0.27	0.27	0.27	0.27	0.27	0.27	0.27	0.27	0.27	0.27
2-2: Electrometer reading in numerator	0.12	0.12	0.12	0.12	0.12	0.12	0.12	0.12	0.12	0.12	0.12	0.12
3: Correction for influence quantities												
3-1: Temperature–pressure correction in denominator												
3-1-1: Instrument error in thermometer	0.10	0.10	0.10	0.10	0.10	0.10	0.10	0.10	0.10	0.10	0.10	0.10
3-1-2: Instrument error in barometer	0.04	0.04	0.04	0.10	0.10	0.10	0.04	0.04	0.04	0.10	0.10	0.10
3-2: Temperature–pressure correction in numerator												
3-2-1: Instrument error in thermometer	0.10	0.10	0.10	0.10	0.10	0.10	0.10	0.10	0.1	0.10	0.10	0.10
3-2-2: Instrument error in barometer	0.04	0.04	0.04	0.10	0.10	0.10	0.04	0.04	0.04	0.10	0.10	0.10
Combined standard uncertainty	0.403	0.420	0.391	0.485	0.491	0.466	0.443	0.433	0.399	0.528	0.512	0.478
Expanded uncertainty (*k* = 2)	0.81	0.84	0.79	0.97	0.99	0.94	0.89	0.87	0.80	1.1	1.1	0.96

Values except for expanded uncertainties (*k* = 2) are given as relative standard uncertainties (*k* = 1). These relative uncertainties except for charge measurement can vary among clinics

Table 9 presents the uncertainties in the PDDs at a depth of 10 cm for the 4-, 6-, and 10-MV photons. Supplementary Tables 13–18 present these budgets. The combined uncertainties (*k* = 1) were approximately 0.40–0.44% and 0.48–0.53% for the realistic and conservative estimates, respectively. The major contributors were the water evaporation, SSD, and charge measurement.

#### OCR

Table 10 presents the uncertainties in the OCRs at 4 cm off axis for the 4-, 6-, and 10-MV photons. Supplementary Tables 19–24 present these budgets. The combined uncertainties (*k* = 1) were approximately 0.44–0.49% and 0.60–0.69% for the realistic and conservative estimates, respectively. The major contributors to these uncertainties were the setting chamber at a depth of 10 cm, SSD, field size, water evaporation, and charge measurement.

**Table 10:** Uncertainties in the measurements of the OCRs at 4 and 5.5 cm off axis from the central axis of the 4-, 6-, and 10-MV photon beams at 10-cm depth

	OCR at 4 cm off axis (Inner region)						OCR at 5.5 cm off axis (Penumbra region)					
	Realistic estimate			Conservative estimate			Realistic estimate			Conservative estimate		
	4 MV	6 MV	10 MV	4 MV	6 MV	10 MV	4 MV	6 MV	10 MV	4 MV	6 MV	10 MV
Component of uncertainty	Value (%)	Value (%)	Value (%)	Value (%)	Value (%)	Value (%)	Value (%)	Value (%)	Value (%)	Value (%)	Value (%)	Value (%)
1: Measurement conditions												
1-1: SSD setting	0.12	0.12	0.12	0.23	0.23	0.23	0.12	0.12	0.12	0.23	0.23	0.23
1-2: Chamber setting on beam axis at 10 cm depth	0.15	0.13	0.11	0.31	0.27	0.23	0.15	0.13	0.11	0.31	0.27	0.23
1-3: Chamber setting at 4 cm/5.5 cm off axis	0.01	0.01	0.01	0.01	0.01	0.01	1.4	1.4	1.3	1.4	1.4	1.3
1-4: Field-size setting												
1-4-1: Chamber reading on beam axis at 10 cm depth	0.07	0.07	0.06	0.14	0.14	0.12	0.07	0.07	0.06	0.14	0.14	0.12
1-4-2: Chamber reading at 4 cm/5.5 cm off axis	0.17	0.15	0.14	0.33	0.29	0.27	7.1	7.0	6.3	14.2	13.9	12.6
1-5: Water evaporation	0.25	0.24	0.18	0.25	0.24	0.18	0.25	0.24	0.18	0.25	0.24	0.18
2: Charge measurement												
2-1: Electrometer reading in denominator	0.27	0.27	0.27	0.27	0.27	0.27	0.27	0.27	0.27	0.27	0.27	0.27
2-2: Electrometer reading in numerator	0.12	0.12	0.12	0.12	0.12	0.12	0.12	0.12	0.12	0.12	0.12	0.12
3: Correction for influence quantities												
3-1: Temperature–pressure correction in denominator												
3-1-1: Instrument error in thermometer	0.10	0.10	0.10	0.10	0.10	0.10	0.10	0.10	0.10	0.10	0.10	0.10
3-1-2: Instrument error in barometer	0.04	0.04	0.04	0.10	0.10	0.10	0.04	0.04	0.04	0.10	0.10	0.10
3-2: Temperature–pressure correction in numerator												
3-2-1: Instrument error in thermometer	0.10	0.10	0.10	0.10	0.10	0.10	0.10	0.10	0.10	0.10	0.10	0.10
3-2-2: Instrument error in barometer	0.04	0.04	0.04	0.10	0.10	0.10	0.04	0.04	0.04	0.10	0.10	0.10
Combined standard uncertainty	0.492	0.474	0.435	0.687	0.648	0.595	7.23	7.11	6.44	14.2	14.0	12.7
Expanded uncertainty (*k* = 2)	0.99	0.95	0.87	1.4	1.3	1.2	15	15	13	29	28	26

Values except for expanded uncertainties (*k* = 2) are given as relative standard uncertainties (*k* = 1). These relative uncertainties except for charge measurement can vary among clinics

Table 10 presents the uncertainties in the OCRs at 5.5 cm off axis for the 4-, 6-, and 10-MV photons. Supplementary Tables 25–30 present these budgets. The combined uncertainties (*k* = 1) were approximately 6.4–7.2% and 13–14% for the realistic and conservative estimates, respectively. The largest uncertainty component was the chamber reading at 5.5 cm off axis owing to the uncertainty in the field-size setting.

### Dose calculations in TPS

Table 11 presents the uncertainties in the calculations of the PDDs for the 4-, 6-, and 10-MV photons in the buildup regions. Supplementary Tables 31–34 present these budgets. The combined uncertainties (*k* = 1) were approximately 0.55–1.4% and 5.8% for the realistic and conservative estimates, respectively.

**Table 11:** Uncertainties in the calculations of the PDDs for the 4-, 6-, and 10-MV photon beams in the buildup and inner regions of the central beam

	Buildup region						Inner region					
	Realistic estimate			Conservative estimate			Realistic estimate			Conservative estimate		
	4 MV	6 MV	10 MV	4 MV	6 MV	10 MV	4 MV	6 MV	10 MV	4 MV	6 MV	10 MV
Component of uncertainty	Value (%)	Value (%)	Value (%)	Value (%)	Value (%)	Value (%)	Value (%)	Value (%)	Value (%)	Value (%)	Value (%)	Value (%)
1: CT number	0.01	0.01	0.01	0.14	0.14	0.14	0.01	0.01	0.01	0.14	0.14	0.14
2: PDD in the buildup/inner region	0.98	0.52	1.4	5.8	5.8	5.8	0.64	0.29	0.69	1.2	1.2	1.2
3: Beam attenuation by treatment couch	0.25	0.18	0.14	0.35	0.35	0.35	0.25	0.18	0.14	0.35	0.35	0.35
Combined standard uncertainty	1.01	0.552	1.39	5.79	5.79	5.79	0.684	0.343	0.707	1.21	1.21	1.21
Expanded uncertainty (*k* = 2)	2.1	1.1	2.8	12	12	12	1.4	0.69	1.5	2.5	2.5	2.5

Values except for expanded uncertainties (*k* = 2) are given as relative standard uncertainties (*k* = 1). These relative uncertainties can vary among clinics

Table 11 presents the uncertainties in the calculations of the PDDs for the 4-, 6-, and 10-MV photons in the inner regions. Supplementary Tables 35–38 present these budgets. The combined uncertainties (*k* = 1) were approximately 0.34–0.71% and 1.2% for the realistic and conservative estimates, respectively.

The major contributor to the uncertainties in the PDD calculations in the buildup and inner regions was the accuracy of those calculations (i.e., ${\delta }_{calc, meas}$ in PDDs).

Table 12 presents the uncertainties of the OCR calculations for the 4-, 6-, and 10-MV photons in the inner regions. Supplementary Tables 39–42 list these budgets. The combined uncertainties (*k* = 1) were approximately 1.4–2.0% and 1.8% for the realistic and conservative estimates, respectively.

**Table 12:** Uncertainties in the calculations of the OCRs for the 4-, 6-, and 10-MV photon beams in the inner and penumbra regions of the beam profile at a depth of 10 cm

	Inner region						Penumbra region					
	Realistic estimate			Conservative estimate			Realistic estimate			Conservative estimate		
	4 MV	6 MV	10 MV	4 MV	6 MV	10 MV	4 MV	6 MV	10 MV	4 MV	6 MV	10 MV
Component of uncertainty	Value (%)	Value (%)	Value (%)	Value (%)	Value (%)	Value (%)	Value (%)	Value (%)	Value (%)	Value (%)	Value (%)	Value (%)
1: CT number	0.01	0.01	0.01	0.14	0.14	0.14	0.01	0.01	0.01	0.14	0.14	0.14
2: OCR in the inner/penumbra region	2.0	1.7	1.4	1.7	1.7	1.7	15.2	15.0	17.6	5.8	5.8	5.8
3: Beam attenuation by treatment couch	0.25	0.18	0.14	0.35	0.35	0.35	0.25	0.18	0.14	0.35	0.35	0.35
Combined standard uncertainty	1.98	1.74	1.39	1.77	1.77	1.77	15.2	15.0	17.6	5.79	5.79	5.79
Expanded uncertainty (*k* = 2)	4.0	3.5	2.8	3.6	3.6	3.6	31	30	36	12	12	12

Values except for expanded uncertainties (*k* = 2) are given as relative standard uncertainties (*k* = 1). These relative uncertainties can vary among clinics

Table 12 presents the uncertainties in the OCR calculations for the 4-, 6-, and 10-MV photons in the penumbra regions. Supplementary Tables 43–46 present these budgets. The combined uncertainties (*k* = 1) in the realistic and conservative estimates were approximately 15–18% and 5.8%, respectively.

The major contributors to the uncertainties in the OCR calculations in the inner and penumbra regions were the accuracy of those calculations (i.e., ${\delta }_{calc, meas}$ in the OCRs).

### Patient setup

Table 13 presents the uncertainties in patient positioning using the ExacTrac X-ray system. Supplementary Tables 47 and 48 present these uncertainty budgets. The expanded uncertainties (*k* = 2) in the realistic and conservative estimates were 0.98 mm and 3.7 mm, respectively.

**Table 13:** Uncertainties in patient positioning with the IGRT system

	Realistic estimate	Conservative estimate
Component of uncertainty	Value (mm)	Value (mm)
1: Image registration	0.22	1.2
2: Coincidence of imaging isocenter and radiation isocenter	0.30	1.2
3: Variation in radiation isocenter due to gantry rotation	0.23	0.58
4: Variation in radiation isocenter due to couch rotation	0.22	0.58
Combined standard uncertainty	0.490	1.83
Expanded uncertainty (*k* = 2)	0.98	3.7

Values except for expanded uncertainties (*k* = 2) are given as standard uncertainties (*k* = 1). These standard uncertainties can vary among clinics

Table 14 presents the uncertainties in the intrafractional head motion under conventionally fractionated irradiation. Supplementary Tables 49 and 50 present these budgets. The expanded uncertainties (*k* = 2) in the realistic and conservative estimates were 3.3 and 3.5 mm, respectively.

**Table 14:** Uncertainties in intrafractional head motion under the conventionally fractionated irradiation

	Realistic estimate	Conservative estimate
Component of uncertainty	Value (mm)	Value (mm)
1: Mean displacement from imaging isocenter	1.06	–
2: Variation in intrafractional motion	1.21	1.73
Combined standard uncertainty	1.61	1.73
Expanded uncertainty (*k* = 2)	3.3	3.5

Values except for expanded uncertainties (*k* = 2) are given as standard uncertainties (*k* = 1). These standard uncertainties can vary among clinics

All uncertainty components presented in Tables 13 and 14 were major contributors to the uncertainties in patient positioning and intrafractional head motion.

### Dosimetric and geometric uncertainties

Table 15 presents the dosimetric uncertainties in the EBRT process for the 4-, 6-, and 10-MV photons. These uncertainties (*k* = 1) in the realistic and conservative estimates were approximately 17–19% and 15–17%, respectively. The major contributors to dosimetric uncertainties were dose calculations and relative measurements in the penumbral regions.

**Table 15:** Dosimetric uncertainties in the EBRT process with the 4-, 6-, and 10-MV photon beams

	Realistic estimate			Conservative estimate		
	4 MV	6 MV	10 MV	4 MV	6 MV	10 MV
Component of uncertainty	Value (%)	Value (%)	Value (%)	Value (%)	Value (%)	Value (%)
1: Reference dosimetry	1.23	1.23	1.22	1.42	1.40	1.39
2: Relative dosimetry^a^	7.27	7.15	6.48	14.3	14.0	12.7
2-1: PDD at buildup	0.403	0.420	0.391	0.485	0.491	0.466
2-2: PDD at inner	0.443	0.433	0.399	0.528	0.512	0.478
2-3: OCR at inner	0.492	0.474	0.435	0.687	0.648	0.595
2-4: OCR at penumbra	7.23	7.11	6.44	14.2	14.0	12.7
3: Dose calculation^b^	15.4	15.1	17.7	8.46	8.46	8.46
3-1: PDD in buildup	1.01	0.552	1.39	5.79	5.79	5.79
3-2: PDD in inner	0.684	0.343	0.707	1.21	1.21	1.21
3-3: OCR in inner	1.98	1.74	1.39	1.77	1.77	1.77
3-4: OCR in penumbra	15.2	15.0	17.6	5.79	5.79	5.79
Combined standard uncertainty	17.1	16.8	18.9	16.7	16.4	15.3
Expanded uncertainty (*k* = 2)	35	34	38	34	33	31

Values except for expanded uncertainties (*k* = 2) are given as relative standard uncertainties (*k* = 1)

^a^Combining components 2-1, 2-2, 2-3, and 2-4

^b^Combining components 3-1, 3-2, 3-3, and 3-4

Table 16 presents the geometric uncertainties. These uncertainties (*k* = 2) in the realistic and conservative estimates were 3.4 and 5.1 mm, respectively. The major contributors were patient positioning and intrafractional head motion.

**Table 16:** Geometric uncertainties in the EBRT process

	Realistic estimate	Conservative estimate
Component of uncertainty	Value (mm)	Value (mm)
1: Patient positioning with the IGRT system	0.490	1.83
2: Intrafractional head motion	1.61	1.73
Combined standard uncertainty	1.68	2.52
Expanded uncertainty (*k* = 2)	3.4	5.1

Values except for expanded uncertainties (*k* = 2) are given as standard uncertainties (*k* = 1)

## Discussion

### Reference dosimetry

The major contributor to the uncertainties in the absorbed dose to water was ${k}_{Q,{Q}_{0}}$ (Table 8). If the ionization chamber was calibrated directly in MV photons [45], these uncertainties (*k* = 1) would be reduced from approximately 1.2–1.4% to 0.74–1.0% (see Supplementary Tables 51–56, which present realistic and conservative uncertainties in the reference dosimetry of the 4-, 6-, and 10-MV photons using the ionization chamber calibrated in MV photons). Medical physicists may use a code of practice for dosimetry using a radiotherapy ionization chamber calibrated in a clinical linac [45] for the reference dosimetry.

The uncertainties (*k* = 1) in the absorbed dose to water were approximately 1.2–1.4% (Table 8). However, in some clinics [46] that did not calibrate their dosimeters over a long period, these uncertainties could be greater than 1.2–1.4%. Medical physicists should calibrate their dosimeters to control uncertainties in the absorbed dose to water.

#### Relative dosimetry

##### PDD

The major contributors to the uncertainties in the PDDs at the buildup and inner regions were the charge measurement, SSD, water evaporation, and setting chamber (Table 9). These contributors would depend on clinical dosimetry practice. Therefore, medical physicists should pay close attention to this practice.

- The uncertainty in the charge measurement was estimated under the standard operating conditions [e.g., the temperature (23 ± 5 °C) and humidity (40 ± 20%)] described in the JSMP Electrometer Guidelines 17 [25]. Deviations from these conditions can result in additional measurement uncertainties. The standard operating conditions should be maintained in treatment rooms.
- The major contributor to the uncertainty in water surface evaporation was the mean displacement after the water level adjustment. The displacement should be checked visually once every few hours during the relative measurements and then adjusted by adding water for at least every 1-mm change in the water level.
- The main component of the uncertainty in the setting chamber was the definition of the origin of the phantom-positioning system, which depends on the user technique. A consistent technique, such as the simple optical method proposed by Das et al. [47], should be adopted for training personnel for setting the origin at the water surface.
- The level of uncertainty in the SSD depends on the localization accuracy of the laser on the lateral walls of linac rooms. This accuracy should be maintained within ± 1 mm rather than within ± 2 mm to reduce this uncertainty (*k* = 1) from 0.23% to 0.12% (see Tables 2 and 9).

##### OCR

The main uncertainty components in the OCRs at the 4 cm off axis were the chamber setting at a depth of 10 cm, SSD, field size, water surface evaporation, and charge measurement (Table 10). In this measurement, as well as in the PDD measurements, the setting chamber, water surface evaporation, and charge measurements should be considered. In addition, the field size should be considered a major uncertainty component in OCR inner measurements. A consistent field size (e.g., with a value of < 1 mm for the sum of the variations in the X- and Y-jaw positions, rather than 2 mm) should be maintained.

The largest uncertainty component in the OCRs at the 5.5 cm off axis was the chamber reading at this off axis (Table 10), owing to the field size uncertainties. Even if the summation of the variations in the X- and Y-jaw positions was 1 mm, the uncertainties (*k* = 1) in the OCRs at the 5.5 cm off axis were approximately 6.4–7.2% (Table 10). The measurements of OCRs in the penumbral regions are challenging and involves high levels of uncertainty. A consistent field size of < 1 mm for the sum of variations in the X- and Y-jaw positions should be maintained, as previously mentioned. Moreover, tolerances for penumbral dose measurements should be set based on the uncertainty of penumbral OCRs.

#### Dose calculations in TPS

The largest components of uncertainties in the calculations of OCRs and PDDs (in the virtual water phantom) were the accuracies of the doses calculated using the TPS. Referring to a publication [48], these accuracies would not depend on the Type B and Type C dose calculation algorithms [49]; these uncertainties might not easily be reduced using an anisotropic analytic algorithm instead of the AXB of the TPS.

The uncertainties in the dose calculations in the penumbral regions were significantly larger than those in the inner regions. This could be because the calculation accuracies of the penumbral doses were significantly lower than those of the inner lateral doses, owing to the high uncertainties in the penumbral dose measurements. Therefore, medical physicists need to control the uncertainties in these measurements, also in the sense of reducing the uncertainties in the penumbral dose calculations.

#### Patient setup

The major contributors to the uncertainties in patient positioning and intrafractional head motion were the uncertainty components presented in Tables 13 and 14. All these contributors should be controlled; therefore, the quality assurances of the image registration accuracy, coincidence of radiation and imaging isocenters, and variations in the radiation isocenter due to gantry and couch rotation should be ensured following implementation guidelines such as AAPM Reports [34, 50] for their quality assurance procedures. In addition, continuous intrafractional motion monitoring should be performed using an effective tool such as a surface-guided radiation therapy system [51, 52].

#### Dosimetric and geometric uncertainties in the EBRT process

The dosimetric uncertainties (*k* = 1) were approximately 15–19% (Table 15). The major contributors were the relative measurements and dose calculations in the penumbra regions; MV photon beam penumbras would have high uncertainties in EBRT. The uncertainties in these measurements and calculations should be reduced.

The geometric uncertainties (*k* = 2) in the realistic and conservative estimates were 3.4 and 5.1 mm, respectively (Table 16). For example, it may be challenging to apply the 3-mm planning target volume (PTV) margin recommended in the guidelines [53] for glioblastoma, which is the most common malignant primary brain tumor. It is not sufficient to barely meet the variation limits used for the conservative estimates in Tables 6 and 7. These limits should be set based on PTV margins used in clinical practice.

### Application of the uncertainties in clinics

ICRU Report 24 [3] indicated that an accuracy of 5% is required for the delivery of the absorbed dose to the target volume. Therefore, each step of EBRT must be performed with an uncertainty of significantly less than 5% such as in the reference dosimetry, relative dosimetry, and dose calculations or 5 mm such as in the patient setup. In the EBRT process evaluated herein, the uncertainties in the relative dosimetry and dose calculations must be reduced to less than 5% (*k* = 1). The uncertainty budgets evaluated herein can be used to determine whether each of the uncertainties in the four steps is significantly lower than 5% or 5 mm. Major contributors to these budgets can also be identified.

Realistic uncertainties (*k* = 2) in the four steps can be applied to set reasonable variation limits (with a confidence level of approximately 95%) for individual institutions. These limits can be set for the reference dosimetry [based on the expanded uncertainties comprising all the uncertainty components presented in Table 8, except for those associated with the calibration of the dosimeter and ${k}_{Q,{Q}_{0}}$], relative dosimetry (based on the expanded uncertainties presented in Tables 9 and 10), dose calculations (based on the expanded uncertainties presented in Tables 11 and 12), patient positioning (based on the expanded uncertainties presented in Table 13), and intrafractional motion (based on the expanded uncertainties presented in Table 14).

### Study limitations

This study has several limitations. First, the uncertainties in the four steps were not evaluated using the various types of radiotherapy equipment (linacs, dosimetry equipment, TPSs, and IGRT systems) used in modern clinics. These uncertainties reflect only examples of conventionally fractionated EBRT for intracranial disease. Second, we did not evaluate uncertainty components (e.g., target volume delineation, organ motion, organ deformations, and tumor- and patient-related changes) related to patients. These evaluations were challenging because their PDFs were not expressed as normal distributions [21]. Third, we did not evaluate uncertainties [54] [e.g., multileaf collimator (MLC) leaf positions, MLC leaf speed, gantry rotational stability, table motion stability, and beam stability (flatness, symmetry, output, dose rate, and segments with low MUs)] associated with intensity-modulated radiotherapy, which would be implemented in many facilities. Finally, we did not evaluate the uncertainty in dose distribution on the TPS. Because of multiple components, such as dose calculation algorithms and parameters (e.g., gantry, couch, collimator, and linac stability) related to the linac, simple statements about the uncertainty in the dose distribution may not easily be made. Future studies on presentation methods for uncertainties related to patients and dose distributions are necessary.

## Conclusion

The uncertainty budgets of the four groups showed the following major contributions to their uncertainties:

- Reference dosimetry: $${k}_{Q,{Q}_{0}}$$
- Relative dosimetry: charge measurements, chamber depth, water evaporation, SSD, and field size
- Dose calculations: dose calculation accuracy
- Patient setup: image registration performed by the IGRT system, coincidence of the imaging and radiation isocenters, variation in the radiation isocenter due to gantry and couch rotation, and intrafractional motion

To reduce the uncertainty in EBRT, these contributions, especially those to the relative dosimetry and dose calculations, should be controlled because the uncertainties of the dose calculations and relative measurements in the penumbral regions exceeded 5%.

## Supplementary Information

Below is the link to the electronic supplementary material.

Supplementary file1 (PDF 1308 KB)

## Data Availability

The authors confirm that the data supporting the findings of this study are available in the article and its supplementary materials.
