# Supplementary material for: Quantification of uncertainties in reference and relative dose measurements, dose calculations, and patient setup in modern external beam radiotherapy
Source: Radiol Phys Technol. 2024 Nov 14;18(1):58–77. doi: 10.1007/s12194-024-00856-0 (PMC11876197; doi:10.1007/s12194-024-00856-0)
Supplement: Supplementary file 1 — Supplementary file1 (PDF 1308 KB) [file 12194_2024_856_MOESM1_ESM.pdf]

Supplementary Table 1. Realistic uncertainty in the reference dosimetry of the 4 MV photon beam.

Component of uncertainty	PDF		Divisor	Standard uncertainty	Sensitivity coefficient	Uncertainty contribution (%)
	Shape	Variation limit/ Standard deviation/ Deflection				
1: Reference conditions						
1-1: SCD setting	Uniform	±1 mm	1.73	0.58 mm	0.2%/mm	0.12
1-2: Chamber setting	Normal	±0.26 mm[a]	1	0.26 mm	0.66%/mm	0.18
1-2-1: Setting origin in phantom	Uniform	±0.4 mm	1.73	0.23 mm	-	
1-2-2: Position accuracy	Uniform	±0.2 mm	1.73	0.12 mm	-	
1-2-3: Position reproducibility	Uniform	±0.1 mm	1.73	0.06 mm	-	
1-3: Field-size setting	Uniform	±1 mm	1.73	0.58 mm	0.20%/mm	0.11
2: Charge measurement	Normal	±0.35%[b]	1	0.35%	1	0.35
2-1: Electrometer	Normal	±0.35%	1	0.35%	-	
2-2: M_{raw} relative to 100 MU	Normal	±0.01%	1	0.01%	-	
3: Long-term stability of ion chamber	Uniform	±0.2%	1.73	0.12%	1	0.12
4: Correction for influence quantities						
4-1: Pressure and temperature						
4-1-1: Instrument error in thermometer	Uniform	±0.5 °C	1.73	0.29 °C	0.34%/°C	0.10
4-1-2: Instrument error in barometer	Uniform	±0.07 kPa	1.73	0.04 kPa	0.99%/kPa	0.04
4-2: Polarity effect						
4-2-1: $\frac{M_+}{M_-}$	Normal	±0.02%	1	0.02%	0.5	0.01
4-3: Ion Recombination						
4-3-1: $\frac{M_1}{M_2}$	Normal	±0.02%	1	0.02%	0.48	0.01
4-4: Humidity	Normal	±0.15%	1	0.15%	1	0.15
5: Calibration of dosimeter						
5-1: Ion chamber	Normal	±1.0%	2	0.50%	1	0.50
5-2: Electrometer	Normal	±0.15%	2	0.075%	1	0.075
6: Determination of k_{Q,Q_0}						
6-1: $\mathrm{TPR}_{20,10}$	Normal	±0.24%[c]	1	0.24%	0.04	0.01
6-1-1: Chamber position at 10-cm depth	Normal	±0.18%	1	0.18%	-	
6-1-2: M_{raw} at 10-cm depth	Normal	±0.01%	1	0.01%	-	
6-1-3: Chamber position at 20-cm depth	Normal	±0.17%	1	0.17%	-	
6-1-4: M_{raw} at 20-cm depth	Normal	±0.01%	1	0.01%	-	
6-2: k_{Q,Q_0}	Normal	±1.0%	1	1.0%	1	1.0
6-3: Difference in $s_{w,air}$	Deflection	0.2%	-	0.2%	1	0.2
Combined standard uncertainty	Normal					1.23
Expanded uncertainty (*k* = 2)						2.5

[a]Combining components 1-2-1, 1-2-2, and 1-2-3.

[b]Combining components 2-1 and 2-2.

[c]Combining components 6-1-1, 6-1-2, 6-1-3, and 6-1-4.

PDF, probability density function; SCD, source-chamber distance; M_{raw}, mean chamber reading; MU, monitor units; M_+ and M_-, mean-chamber readings obtained at polarizing voltages of −300 and +300 V, respectively; M_1 and M_2, mean-collected charges obtained at polarizing voltages of +300 and +100 V, respectively; k_{Q,Q_0}, beam quality conversion factor; $\mathrm{TPR}_{20,10}$, tissue–phantom ratio in water at depths of 20 and 10 cm; $s_{w,air}$, water-to-air stopping-power ratio

Supplementary Table 2. Realistic uncertainty in the reference dosimetry of the 6 MV photon beam.

Component of uncertainty	PDF		Divisor	Standard uncertainty	Sensitivity coefficient	Uncertainty contribution (%)
	Shape	Variation limit/ Standard deviation/ Deflection				
1: Reference conditions						
1-1: SCD setting	Uniform	±1 mm	1.73	0.58 mm	0.2%/mm	0.12
1-2: Chamber setting	Normal	±0.26 mm[a]	1	0.26 mm	0.57%/mm	0.15
1-2-1: Setting origin in phantom	Uniform	±0.4 mm	1.73	0.23 mm	-	
1-2-2: Position accuracy	Uniform	±0.2 mm	1.73	0.12 mm	-	
1-2-3: Position reproducibility	Uniform	±0.1 mm	1.73	0.06 mm	-	
1-3: Field-size setting	Uniform	±1 mm	1.73	0.58 mm	0.15%/mm	0.09
2: Charge measurement	Normal	±0.35%[b]	1	0.35%	1	0.35
2-1: Electrometer	Normal	±0.35%	1	0.35%	-	
2-2: M_{raw} relative to 100 MU	Normal	±0.01%	1	0.01%	-	
3: Long-term stability of ion chamber	Uniform	±0.2%	1.73	0.12%	1	0.12
4: Correction for influence quantities						
4-1: Pressure and temperature						
4-1-1: Instrument error in thermometer	Uniform	±0.5 ℃	1.73	0.29 ℃	0.34%/℃	0.10
4-1-2: Instrument error in barometer	Uniform	±0.07 kPa	1.73	0.04 kPa	0.99%/kPa	0.04
4-2: Polarity effect						
4-2-1: $\frac{M_+}{M_-}$	Normal	±0.01%	1	0.01%	0.5	0.006
4-3: Ion Recombination						
4-3-1: $\frac{M_1}{M_2}$	Normal	±0.01%	1	0.01%	0.48	0.005
4-4: Humidity	Normal	±0.15%	1	0.15%	1	0.15
5: Calibration of dosimeter						
5-1: Ion chamber	Normal	±1.0%	2	0.50%	1	0.50
5-2: Electrometer	Normal	±0.15%	2	0.075%	1	0.075
6: Determination of k_{Q,Q_0}						
6-1: $\mathrm{TPR}_{20,10}$	Normal	±0.22%[c]	1	0.22%	0.09	0.02
6-1-1: Chamber position at 10-cm depth	Normal	±0.15%	1	0.15%	-	
6-1-2: M_{raw} at 10-cm depth	Normal	±0.01%	1	0.01%	-	
6-1-3: Chamber position at 20-cm depth	Normal	±0.16%	1	0.16%	-	
6-1-4: M_{raw} at 20-cm depth	Normal	±0.007%	1	0.007%	-	
6-2: k_{Q,Q_0}	Normal	±1.0%	1	1.0%	1	1.0
6-3: Difference in $s_{w,air}$	Deflection	0.2%	-	0.2%	1	0.2
Combined standard uncertainty	Normal					1.23
Expanded uncertainty (k = 2)						2.5

[a]Combining components 1-2-1, 1-2-2, and 1-2-3.

[b]Combining components 2-1 and 2-2.

[c]Combining components 6-1-1, 6-1-2, 6-1-3, and 6-1-4.

PDF, probability density function; SCD, source-chamber distance; M_{raw}, mean chamber reading; MU, monitor units; M_+ and M_-, mean-chamber readings obtained at polarizing voltages of -300 and $+300$ V, respectively; M_1 and M_2, mean-collected charges obtained at polarizing voltages of $+300$ and $+100$ V, respectively; k_{Q,Q_0}, beam quality conversion factor; $\mathrm{TPR}_{20,10}$, tissue–phantom ratio in water at depths of 20 and 10 cm; $s_{w,air}$, water-to-air stopping-power ratio

Supplementary Table 3. Realistic uncertainty in the reference dosimetry of the 10 MV photon beam.

Component of uncertainty	PDF		Divisor	Standard uncertainty	Sensitivity coefficient	Uncertainty contribution (%)
	Shape	Variation limit/ Standard deviation/ Deflection				
1: Reference conditions						
1-1: SCD setting	Uniform	±1 mm	1.73	0.58 mm	0.2%/mm	0.12
1-2: Chamber setting	Normal	±0.26 mm[a]	1	0.26 mm	0.47%/mm	0.13
1-2-1: Setting origin in phantom	Uniform	±0.4 mm	1.73	0.23 mm	-	
1-2-2: Position accuracy	Uniform	±0.2 mm	1.73	0.12 mm	-	
1-2-3: Position reproducibility	Uniform	±0.1 mm	1.73	0.06 mm	-	
1-3: Field-size setting	Uniform	±1 mm	1.73	0.58 mm	0.11%/mm	0.07
2: Charge measurement	Normal	±0.35%[b]	1	0.35%	1	0.35
2-1: Electrometer	Normal	±0.35%	1	0.35%	-	
2-2: M_{raw} relative to 100 MU	Normal	±0.005%	1	0.005%	-	
3: Long-term stability of ion chamber	Uniform	±0.2%	1.73	0.12%	1	0.12
4: Correction for influence quantities						
4-1: Pressure and temperature						
4-1-1: Instrument error in thermometer	Uniform	±0.5 °C	1.73	0.29 °C	0.34%/°C	0.10
4-1-2: Instrument error in barometer	Uniform	±0.07 kPa	1.73	0.04 kPa	0.99%/kPa	0.04
4-2: Polarity effect						
4-2-1: $\frac{M_+}{M_-}$	Normal	±0.005%	1	0.005%	0.5	0.003
4-3: Ion Recombination						
4-3-1: $\frac{M_1}{M_2}$	Normal	±0.008%	1	0.008%	0.48	0.004
4-4: Humidity	Normal	±0.15%	1	0.15%	1	0.15
5: Calibration of dosimeter						
5-1: Ion chamber	Normal	±1.0%	2	0.50%	1	0.50
5-2: Electrometer	Normal	±0.15%	2	0.075%	1	0.075
6: Determination of k_{Q,Q_0}						
6-1: $\mathrm{TPR}_{20,10}$	Normal	±0.18%[c]	1	0.18%	0.18	0.03
6-1-1: Chamber position at 10-cm depth	Normal	±0.13%	1	0.13%	-	
6-1-2: M_{raw} at 10-cm depth	Normal	±0.005%	1	0.005%	-	
6-1-3: Chamber position at 20-cm depth	Normal	±0.13%	1	0.13%	-	
6-1-4: M_{raw} at 20-cm depth	Normal	±0.005%	1	0.005%	-	
6-2: k_{Q,Q_0}	Normal	±1.0%	1	1.0%	1	1.0
6-3: Difference in $s_{w,air}$	Deflection	0.2%	-	0.2%	1	0.2
Combined standard uncertainty	Normal					1.22
Expanded uncertainty (k = 2)						2.5

[a]Combining components 1-2-1, 1-2-2, and 1-2-3.

[b]Combining components 2-1 and 2-2.

[c]Combining components 6-1-1, 6-1-2, 6-1-3, and 6-1-4.

PDF, probability density function; SCD, source-chamber distance; M_{raw}, mean chamber reading; MU, monitor units; M_+ and M_-, mean-chamber readings obtained at polarizing voltages of −300 and +300 V, respectively; M_1 and M_2, mean-collected charges obtained at polarizing voltages of +300 and +100 V, respectively; k_{Q,Q_0}, beam quality conversion factor; $\mathrm{TPR}_{20,10}$, tissue–phantom ratio in water at depths of 20 and 10 cm; $s_{w,air}$, water-to-air stopping-power ratio

Supplementary Table 4. Conservative uncertainty in the reference dosimetry of the 4 MV photon beam.

Component of uncertainty	PDF		Divisor	Standard uncertainty	Sensitivity coefficient	Uncertainty contribution (%)
	Shape	Variation limit/ Standard deviation/ Deflection				
1: Reference conditions						
1-1: SCD setting	Uniform	±2 mm	1.73	1.2 mm	0.2%/mm	0.23
1-2: Chamber setting	Normal	±0.5 mm	1	0.5 mm	0.66%/mm	0.33
1-3: Field-size setting	Uniform	±2 mm	1.73	1.2 mm	0.20%/mm	0.23
2: Charge measurement	Normal	±0.6%	1	0.6%	1	0.6
3: Long-term stability of ion chamber	Normal	±0.3%	1	0.3%	1	0.3
4: Correction for influence quantities						
4-1: Pressure and temperature						
4-1-1: Instrument error in thermometer	Normal	±0.3 ℃	1	0.3 ℃	0.34%/℃	0.10
4-1-2: Instrument error in barometer	Normal	±0.1 kPa	1	0.1 kPa	0.99%/kPa	0.10
4-2: Polarity effect	Normal	±0.05%	1	0.05%	1	0.05
4-3: Ion Recombination	Normal	±0.10%	1	0.10%	1	0.10
4-4: Humidity	Normal	±0.15%	1	0.15%	1	0.15
5: Calibration of dosimeter						
5-1: Ion chamber	Normal	±0.49%	1	0.49%	1	0.49
5-2: Electrometer	Normal	±0.09%	1	0.09%	1	0.09
6: Determination of k_{Q,Q_0}						
6-1: $\mathrm{TPR}_{20,10}$	Normal	±0.46%[a]	1	0.46%	0.04	0.02
6-1-1: Chamber position at 10-cm depth	Normal	±0.33%	1	0.33%	-	
6-1-2: M_{raw} at 10-cm depth	Normal	±0.05%	1	0.05%	-	
6-1-3: Chamber position at 20-cm depth	Normal	±0.31%	1	0.31%	-	
6-1-4: M_{raw} at 20-cm depth	Normal	±0.05%	1	0.05%	-	
6-2: k_{Q,Q_0}	Normal	±1.0%	1	1.0%	1	1.0
6-3: Difference in $s_{w,air}$	Deflection	0.2%	-	0.2%	1	0.2
Combined standard uncertainty	Normal					1.42
Expanded uncertainty (k = 2)						2.9

[a]Combining components 6-1-1, 6-1-2, 6-1-3, and 6-1-4.

PDF, probability density function; SCD, source-chamber distance; k_{Q,Q_0}, beam quality conversion factor; $\mathrm{TPR}_{20,10}$, tissue–phantom ratio in water at depths of 20 and 10 cm; M_{raw}, mean chamber reading; $s_{w,air}$, water-to-air stopping-power ratio

Supplementary Table 5. Conservative uncertainty in the reference dosimetry of the 6 MV photon beam.

Component of uncertainty	PDF		Divisor	Standard uncertainty	Sensitivity coefficient	Uncertainty contribution (%)
	Shape	Variation limit/ Standard deviation/ Deflection				
1: Reference conditions						
1-1: SCD setting	Uniform	±2 mm	1.73	1.2 mm	0.2%/mm	0.23
1-2: Chamber setting	Normal	±0.5 mm	1	0.5 mm	0.57%/mm	0.29
1-3: Field-size setting	Uniform	±2 mm	1.73	1.2 mm	0.15%/mm	0.17
2: Charge measurement	Normal	±0.6%	1	0.6%	1	0.6
3: Long-term stability of ion chamber	Normal	±0.3%	1	0.3%	1	0.3
4: Correction for influence quantities						
4-1: Pressure and temperature						
4-1-1: Instrument error in thermometer	Normal	±0.3 ℃	1	0.3 ℃	0.34%/℃	0.10
4-1-2: Instrument error in barometer	Normal	±0.1 kPa	1	0.1 kPa	0.99%/kPa	0.10
4-2: Polarity effect	Normal	±0.05%	1	0.05%	1	0.05
4-3: Ion Recombination	Normal	±0.10%	1	0.10%	1	0.10
4-4: Humidity	Normal	±0.15%	1	0.15%	1	0.15
5: Calibration of dosimeter						
5-1: Ion chamber	Normal	±0.49%	1	0.49%	1	0.49
5-2: Electrometer	Normal	±0.09%	1	0.09%	1	0.09
6: Determination of k_{Q,Q_0}						
6-1: $\mathrm{TPR}_{20,10}$	Normal	±0.42%[a]	1	0.42%	0.09	0.04
6-1-1: Chamber position at 10-cm depth	Normal	±0.29%	1	0.29%	-	
6-1-2: M_{raw} at 10-cm depth	Normal	±0.05%	1	0.05%	-	
6-1-3: Chamber position at 20-cm depth	Normal	±0.30%	1	0.30%	-	
6-1-4: M_{raw} at 20-cm depth	Normal	±0.05%	1	0.05%	-	
6-2: k_{Q,Q_0}	Normal	±1.0%	1	1.0%	1	1.0
6-3: Difference in $s_{w,air}$	Deflection	0.20%	-	0.2%	1	0.2
Combined standard uncertainty	Normal					1.40
Expanded uncertainty (k = 2)						2.8

[a]Combining components 6-1-1, 6-1-2, 6-1-3, and 6-1-4.

PDF, probability density function; SCD, source-chamber distance; k_{Q,Q_0}, beam quality conversion factor; $\mathrm{TPR}_{20,10}$, tissue–phantom ratio in water at depths of 20 and 10 cm; M_{raw}, mean chamber reading; $s_{w,air}$, water-to-air stopping-power ratio

Supplementary Table 6. Conservative uncertainty in the reference dosimetry of the 10 MV photon beam.

Component of uncertainty	PDF		Divisor	Standard uncertainty	Sensitivity coefficient	Uncertainty contribution (%)
	Shape	Variation limit/ Standard deviation/ Deflection				
1: Reference conditions						
1-1: SCD setting	Uniform	±2 mm	1.73	1.2 mm	0.2%/mm	0.23
1-2: Chamber setting	Normal	±0.5 mm	1	0.5 mm	0.47%/mm	0.24
1-3: Field-size setting	Uniform	±2 mm	1.73	1.2 mm	0.11%/mm	0.13
2: Charge measurement	Normal	±0.6%	1	0.6%	1	0.6
3: Long-term stability of ion chamber	Normal	±0.3%	1	0.3%	1	0.3
4: Correction for influence quantities						
4-1: Pressure and temperature						
4-1-1: Instrument error in thermometer	Normal	±0.3 ℃	1	0.3 ℃	0.34%/℃	0.10
4-1-2: Instrument error in barometer	Normal	±0.1 kPa	1	0.1 kPa	0.99%/kPa	0.10
4-2: Polarity effect	Normal	±0.05%	1	0.05%	1	0.05
4-3: Ion Recombination	Normal	±0.10%	1	0.10%	1	0.10
4-4: Humidity	Normal	±0.15%	1	0.15%	1	0.15
5: Calibration of dosimeter						
5-1: Ion chamber	Normal	±0.49%	1	0.49%	1	0.49
5-2: Electrometer	Normal	±0.09%	1	0.09%	1	0.09
6: Determination of k_{Q,Q_0}						
6-1: $\mathrm{TPR}_{20,10}$	Normal	±0.35%[a]	1	0.35%	0.18	0.06
6-1-1: Chamber position at 10-cm depth	Normal	±0.24%	1	0.24%	-	
6-1-2: M_{raw} at 10-cm depth	Normal	±0.05%	1	0.05%	-	
6-1-3: Chamber position at 20-cm depth	Normal	±0.24%	1	0.24%	-	
6-1-4: M_{raw} at 20-cm depth	Normal	±0.05%	1	0.05%	-	
6-2: k_{Q,Q_0}	Normal	±1.0%	1	1.0%	1	1.0
6-3: Difference in $s_{w,air}$	Deflection	0.2%	-	0.2%	1	0.2
Combined uncertainty	Normal					1.39
Expanded uncertainty (k = 2)						2.8

[a]Combining components 6-1-1, 6-1-2, 6-1-3, and 6-1-4.

PDF, probability density function; SCD, source-chamber distance; k_{Q,Q_0}, beam quality conversion factor; $\mathrm{TPR}_{20,10}$, tissue–phantom ratio in water at depths of 20 and 10 cm; M_{raw}, mean chamber reading; $s_{w,air}$, water-to-air stopping-power ratio

Supplementary Table 7. Realistic uncertainty in the measurement of the PDD in the buildup region (at 0.6-cm depth) on the central axis of the 4 MV photon beam.

Component of uncertainty	PDF		Divisor	Standard uncertainty	Sensitivity coefficient	Uncertainty contribution (%)
	Shape	Variation limit/ Standard deviation/ Deflection				
1: Measurement conditions						
1-1: SSD setting	Uniform	±1 mm	1.73	0.58 mm	0.2%/mm	0.12
1-2: Setting chamber at depth of maximum dose	Normal	±0.24 mm[a]	1	0.24 mm	0.22%/mm	0.05
1-2-1: Setting origin in phantom	Uniform	±0.4 mm	1.73	0.23 mm	-	
1-2-2: Reproducibility of movement distance	Uniform	±0.1 mm	1.73	0.06 mm	-	
1-3: Setting chamber at 0.6-cm depth						
1-3-1: Reproducibility of movement distance	Uniform	±0.1 mm	1.73	0.06 mm	2.8%/mm	0.16
1-4: Field-size setting	Uniform	±1 mm	1.73	0.58 mm	0.08%/mm	0.04
1-5: Water evaporation	Normal	±0.62 mm[b]	1	0.62 mm	0.15%/mm	0.10
1-5-1: Water level adjustment among users	Uniform	±0.4 mm	1.73	0.23 mm	-	
1-5-2: Mean displacement after water level adjustment	Deflection	0.5 mm	-	0.5 mm	-	
1-5-3: Variation in water level	Uniform	±0.5 mm	1.73	0.29 mm	-	
2: Charge measurement						
2-1: Electrometer reading in denominator						
2-1-1: Display resolution	Normal	±0.058%	1	0.058%	1	0.058
2-1-2: Repeatability	Normal	±0.1%	1	0.1%	1	0.1
2-1-3: Zero drift	Normal	±0.058%	1	0.058%	1	0.058
2-1-4: Non-linearity	Normal	±0.12%	1	0.12%	1	0.12
2-1-5: Response to pulsed beam from a linac	Normal	±0.12%	1	0.12%	1	0.12
2-1-6: Stabilization time	Normal	±0.12%	1	0.12%	1	0.12
2-1-7: Mains voltage fluctuation during measurement	Normal	±0.12%	1	0.12%	1	0.12
2-1-8: Elapsed timer	Normal	±0.01%	1	0.01%	1	0.01
2-2: Electrometer reading in numerator						
2-2-1: Repeatability	Normal	±0.1%	1	0.1%	1	0.1
2-2-2: Zero drift	Normal	±0.058%	1	0.058%	1	0.058
3: Correction for influence quantities						
3-1: Temperature-pressure correction in denominator						
3-1-1: Instrument error in thermometer	Uniform	±0.5 ℃	1.73	0.29 ℃	0.34%/℃	0.10
3-1-2: Instrument error in barometer	Uniform	±0.07 kPa	1.73	0.04 kPa	0.99%/kPa	0.04
3-2: Temperature-pressure correction in numerator						
3-2-1: Instrument error in thermometer	Uniform	±0.5 ℃	1.73	0.29 ℃	0.34%/℃	0.10
3-2-2: Instrument error in barometer	Uniform	±0.07 kPa	1.73	0.04 kPa	0.99%/kPa	0.04
Combined uncertainty	Normal					0.403
Expanded uncertainty (k = 2)						0.81

[a]Combining components 1-2-1 and 1-2-2.

[b]Combining components 1-5-1, 1-5-2, and 1-5-3.

PDF, probability density function; SSD, source to surface distance

Supplementary Table 8. Realistic uncertainty in the measurement of the PDD in the buildup region (at 0.7-cm depth) on the central axis of the 6 MV photon beam.

Component of uncertainty	PDF		Divisor	Standard uncertainty	Sensitivity coefficient	Uncertainty contribution (%)
	Shape	Variation limit/ Standard deviation/ Deflection				
1: Measurement conditions						
1-1: SSD setting	Uniform	±1 mm	1.73	0.58 mm	0.2%/mm	0.12
1-2: Setting chamber at depth of maximum dose	Normal	±0.24 mm[a]	1	0.24 mm	0.06%/mm	0.02
1-2-1: Setting origin in phantom	Uniform	±0.4 mm	1.73	0.23 mm	-	
1-2-2: Reproducibility of movement distance	Uniform	±0.1 mm	1.73	0.06 mm	-	
1-3: Setting chamber at 0.7-cm depth						
1-3-1: Reproducibility of movement distance	Uniform	±0.1 mm	1.73	0.06 mm	3.2%/mm	0.19
1-4: Field-size setting	Uniform	±1 mm	1.73	0.58 mm	0.08%/mm	0.04
1-5: Water evaporation	Normal	±0.62 mm[b]	1	0.62 mm	0.21%/mm	0.13
1-5-1: Water level adjustment among users	Uniform	±0.4 mm	1.73	0.23 mm	-	
1-5-2: Mean displacement after water level adjustment	Deflection	0.5 mm	-	0.5 mm	-	
1-5-3: Variation in water level	Uniform	±0.5 mm	1.73	0.29 mm	-	
2: Charge measurement						
2-1: Electrometer reading in denominator						
2-1-1: Display resolution	Normal	±0.058%	1	0.058%	1	0.058
2-1-2: Repeatability	Normal	±0.1%	1	0.1%	1	0.1
2-1-3: Zero drift	Normal	±0.058%	1	0.058%	1	0.058
2-1-4: Non-linearity	Normal	±0.12%	1	0.12%	1	0.12
2-1-5: Response to pulsed beam from a linac	Normal	±0.12%	1	0.12%	1	0.12
2-1-6: Stabilization time	Normal	±0.12%	1	0.12%	1	0.12
2-1-7: Mains voltage fluctuation during measurement	Normal	±0.12%	1	0.12%	1	0.12
2-1-8: Elapsed timer	Normal	±0.01%	1	0.01%	1	0.01
2-2: Electrometer reading in numerator						
2-2-1: Repeatability	Normal	±0.1%	1	0.1%	1	0.1
2-2-2: Zero drift	Normal	±0.058%	1	0.058%	1	0.058
3: Correction for influence quantities						
3-1: Temperature-pressure correction in denominator						
3-1-1: Instrument error in thermometer	Uniform	±0.5 ℃	1.73	0.29 ℃	0.34%/℃	0.10
3-1-2: Instrument error in barometer	Uniform	±0.07 kPa	1.73	0.04 kPa	0.99%/kPa	0.04
3-2: Temperature-pressure correction in numerator						
3-2-1: Instrument error in thermometer	Uniform	±0.5 ℃	1.73	0.29 ℃	0.34%/℃	0.10
3-2-2: Instrument error in barometer	Uniform	±0.07 kPa	1.73	0.04 kPa	0.99%/kPa	0.04
Combined uncertainty	Normal					0.420
Expanded uncertainty (k = 2)						0.84

[a]Combining components 1-2-1 and 1-2-2.

[b]Combining components 1-5-1, 1-5-2, and 1-5-3.

PDF, probability density function; SSD, source to surface distance

Supplementary Table 9. Realistic uncertainty in the measurement of the PDD in the buildup region (at 1.2-cm depth) on the central axis of the 10 MV photon beam

Component of uncertainty	PDF		Divisor	Standard uncertainty	Sensitivity coefficient	Uncertainty contribution (%)
	Shape	Variation limit/ Standard deviation/ Deflection				
1: Measurement conditions						
1-1: SSD setting	Uniform	±1 mm	1.73	0.58 mm	0.2%/mm	0.12
1-2: Setting chamber at depth of maximum dose	Normal	±0.24 mm[a]	1	0.24 mm	0.09%/mm	0.02
1-2-1: Setting origin in phantom	Uniform	±0.4 mm	1.73	0.23 mm	-	
1-2-2: Reproducibility of movement distance	Uniform	±0.1 mm	1.73	0.06 mm	-	
1-3: Setting chamber at 1.2-cm depth						
1-3-1: Reproducibility of movement distance	Uniform	±0.1 mm	1.73	0.06 mm	1.7%/mm	0.10
1-4: Field-size setting	Uniform	±1 mm	1.73	0.58 mm	0.08%/mm	0.04
1-5: Water evaporation	Normal	±0.62 mm[b]	1	0.62 mm	0.21%/mm	0.13
1-5-1: Water level adjustment among users	Uniform	±0.4 mm	1.73	0.23 mm	-	
1-5-2: Mean displacement after water level adjustment	Deflection	0.5 mm	-	0.5 mm	-	
1-5-3: Variation in water level	Uniform	±0.5 mm	1.73	0.29 mm	-	
2: Charge measurement						
2-1: Electrometer reading in denominator						
2-1-1: Display resolution	Normal	±0.058%	1	0.058%	1	0.058
2-1-2: Repeatability	Normal	±0.1%	1	0.1%	1	0.1
2-1-3: Zero drift	Normal	±0.058%	1	0.058%	1	0.058
2-1-4: Non-linearity	Normal	±0.12%	1	0.12%	1	0.12
2-1-5: Response to pulsed beam from a linac	Normal	±0.12%	1	0.12%	1	0.12
2-1-6: Stabilization time	Normal	±0.12%	1	0.12%	1	0.12
2-1-7: Mains voltage fluctuation during measurement	Normal	±0.12%	1	0.12%	1	0.12
2-1-8: Elapsed timer	Normal	±0.01%	1	0.01%	1	0.01
2-2: Electrometer reading in numerator						
2-2-1: Repeatability	Normal	±0.1%	1	0.1%	1	0.1
2-2-2: Zero drift	Normal	±0.058%	1	0.058%	1	0.058
3: Correction for influence quantities						
3-1: Temperature-pressure correction in denominator						
3-1-1: Instrument error in thermometer	Uniform	±0.5 ℃	1.73	0.29 ℃	0.34%/℃	0.10
3-1-2: Instrument error in barometer	Uniform	±0.07 kPa	1.73	0.04 kPa	0.99%/kPa	0.04
3-2: Temperature-pressure correction in numerator						
3-2-1: Instrument error in thermometer	Uniform	±0.5 ℃	1.73	0.29 ℃	0.34%/℃	0.10
3-2-2: Instrument error in barometer	Uniform	±0.07 kPa	1.73	0.04 kPa	0.99%/kPa	0.04
Combined uncertainty	Normal					0.391
Expanded uncertainty (k = 2)						0.79

[a]Combining components 1-2-1 and 1-2-2.

[b]Combining components 1-5-1, 1-5-2, and 1-5-3.

PDF, probability density function; SSD, source to surface distance

Supplementary Table 10. Conservative uncertainty in the measurement of the PDD in the buildup region (at 0.6-cm depth) on the central axis of the 4 MV photon beam.

Component of uncertainty	PDF		Divisor	Standard uncertainty	Sensitivity coefficient	Uncertainty contribution (%)
	Shape	Variation limit/ Standard deviation/ Deflection				
1: Measurement conditions						
1-1: SSD setting	Uniform	±2 mm	1.73	1.2 mm	0.2%/mm	0.23
1-2: Setting chamber at depth of maximum dose	Normal	±0.5 mm	1	0.5 mm	0.22%/mm	0.11
1-3: Setting chamber at 0.6-cm depth						
1-3-1: Accuracy of movement distance	Uniform	±0.1 mm	1.73	0.06 mm	2.8%/mm	0.16
1-4: Field-size setting	Uniform	±2 mm	1.73	1.2 mm	0.08%/mm	0.09
1-5: Water evaporation	Normal	±0.62 mm[a]	1	0.62 mm	0.15%/mm	0.10
1-5-1: Water level adjustment among users	Uniform	±0.4 mm	1.73	0.23 mm	-	
1-5-2: Mean displacement after water level adjustment	Deflection	0.5 mm	-	0.5 mm	-	
1-5-3: Variation in water level	Uniform	±0.5 mm	1.73	0.29 mm	-	
2: Charge measurement						
2-1: Electrometer reading in denominator						
2-1-1: Display resolution	Normal	±0.058%	1	0.058%	1	0.058
2-1-2: Repeatability	Normal	±0.1%	1	0.1%	1	0.1
2-1-3: Zero drift	Normal	±0.058%	1	0.058%	1	0.058
2-1-4: Non-linearity	Normal	±0.12%	1	0.12%	1	0.12
2-1-5: Response to pulsed beam from a linac	Normal	±0.12%	1	0.12%	1	0.12
2-1-6: Stabilization time	Normal	±0.12%	1	0.12%	1	0.12
2-1-7: Mains voltage fluctuation during measurement	Normal	±0.12%	1	0.12%	1	0.12
2-1-8: Elapsed timer	Normal	±0.01%	1	0.01%	1	0.01
2-2: Electrometer reading in numerator						
2-2-1: Repeatability	Normal	±0.1%	1	0.1%	1	0.1
2-2-2: Zero drift	Normal	±0.058%	1	0.058%	1	0.058
3: Correction for influence quantities						
3-1: Temperature-pressure correction in denominator						
3-1-1: Instrument error in thermometer	Normal	±0.3 ℃	1	0.3 ℃	0.34%/℃	0.10
3-1-2: Instrument error in barometer	Normal	±0.1 kPa	1	0.1 kPa	0.99%/kPa	0.10
3-2: Temperature-pressure correction in numerator						
3-2-1: Instrument error in thermometer	Normal	±0.3 ℃	1	0.3 ℃	0.34%/℃	0.10
3-2-2: Instrument error in barometer	Normal	±0.1 kPa	1	0.1 kPa	0.99%/kPa	0.10
Combined uncertainty	Normal					0.485
Expanded uncertainty (k = 2)						0.97

[a]Combining components 1-5-1, 1-5-2, and 1-5-3.

PDF, probability density function; SSD, source to surface distance

Supplementary Table 11. Conservative uncertainty in the measurement of the PDD in the buildup region (at 0.7-cm depth) on the central axis of the 6 MV photon beam.

Component of uncertainty	PDF		Divisor	Standard uncertainty	Sensitivity coefficient	Uncertainty contribution (%)
	Shape	Variation limit/ Standard deviation/ Deflection				
1: Measurement conditions						
1-1: SSD setting	Uniform	±2 mm	1.73	1.2 mm	0.2%/mm	0.23
1-2: Setting chamber at depth of maximum dose	Normal	±0.5 mm	1	0.5 mm	0.06%/mm	0.03
1-3: Setting chamber at 0.7-cm depth						
1-3-1: Accuracy of movement distance	Uniform	±0.1 mm	1.73	0.06 mm	3.2%/mm	0.19
1-4: Field-size setting	Uniform	±2 mm	1.73	1.2 mm	0.08%/mm	0.09
1-5: Water evaporation	Normal	±0.62 mm[a]	1	0.62 mm	0.21%/mm	0.13
1-5-1: Water level adjustment among users	Uniform	±0.4 mm	1.73	0.23 mm	-	
1-5-2: Mean displacement after water level adjustment	Deflection	0.5 mm	-	0.5 mm	-	
1-5-3: Variation in water level	Uniform	±0.5 mm	1.73	0.29 mm	-	
2: Charge measurement						
2-1: Electrometer reading in denominator						
2-1-1: Display resolution	Normal	±0.058%	1	0.058%	1	0.058
2-1-2: Repeatability	Normal	±0.1%	1	0.1%	1	0.1
2-1-3: Zero drift	Normal	±0.058%	1	0.058%	1	0.058
2-1-4: Non-linearity	Normal	±0.12%	1	0.12%	1	0.12
2-1-5: Response to pulsed beam from a linac	Normal	±0.12%	1	0.12%	1	0.12
2-1-6: Stabilization time	Normal	±0.12%	1	0.12%	1	0.12
2-1-7: Mains voltage fluctuation during measurement	Normal	±0.12%	1	0.12%	1	0.12
2-1-8: Elapsed timer	Normal	±0.01%	1	0.01%	1	0.01
2-2: Electrometer reading in numerator						
2-2-1: Repeatability	Normal	±0.1%	1	0.1%	1	0.1
2-2-2: Zero drift	Normal	±0.058%	1	0.058%	1	0.058
3: Correction for influence quantities						
3-1: Temperature-pressure correction in denominator						
3-1-1: Instrument error in thermometer	Normal	±0.3 ℃	1	0.3 ℃	0.34%/℃	0.10
3-1-2: Instrument error in barometer	Normal	±0.1 kPa	1	0.1 kPa	0.99%/kPa	0.10
3-2: Temperature-pressure correction in numerator						
3-2-1: Instrument error in thermometer	Normal	±0.3 ℃	1	0.3 ℃	0.34%/℃	0.10
3-2-2: Instrument error in barometer	Normal	±0.1 kPa	1	0.1 kPa	0.99%/kPa	0.10
Combined uncertainty	Normal					0.491
Expanded uncertainty (k = 2)						0.99

[a]Combining components 1-5-1, 1-5-2, and 1-5-3.

PDF, probability density function; SSD, source to surface distance

Supplementary Table 12. Conservative uncertainty in the measurement of the PDD in the buildup region (at 1.2-cm depth) on the central axis of the 10 MV photon beam.

Component of uncertainty	PDF		Divisor	Standard uncertainty	Sensitivity coefficient	Uncertainty contribution (%)
	Shape	Variation limit/ Standard deviation/ Deflection				
1: Measurement conditions						
1-1: SSD setting	Uniform	±2 mm	1.73	1.2 mm	0.2%/mm	0.23
1-2: Setting chamber at depth of maximum dose	Normal	±0.5 mm	1	0.5 mm	0.09%/mm	0.04
1-3: Setting chamber at 1.2-cm depth						
1-3-1: Accuracy of movement distance	Uniform	±0.1 mm	1.73	0.06 mm	1.7%/mm	0.10
1-4: Field-size setting	Uniform	±2 mm	1.73	1.2 mm	0.08%/mm	0.09
1-5: Water evaporation	Normal	±0.62 mm[a]	1	0.62 mm	0.21%/mm	0.13
1-5-1: Water level adjustment among users	Uniform	±0.4 mm	1.73	0.23 mm	-	
1-5-2: Mean displacement after water level adjustment	Deflection	0.5 mm	-	0.5 mm	-	
1-5-3: Variation in water level	Uniform	±0.5 mm	1.73	0.29 mm	-	
2: Charge measurement						
2-1: Electrometer reading in denominator						
2-1-1: Display resolution	Normal	±0.058%	1	0.058%	1	0.058
2-1-2: Repeatability	Normal	±0.1%	1	0.1%	1	0.1
2-1-3: Zero drift	Normal	±0.058%	1	0.058%	1	0.058
2-1-4: Non-linearity	Normal	±0.12%	1	0.12%	1	0.12
2-1-5: Response to pulsed beam from a linac	Normal	±0.12%	1	0.12%	1	0.12
2-1-6: Stabilization time	Normal	±0.12%	1	0.12%	1	0.12
2-1-7: Mains voltage fluctuation during measurement	Normal	±0.12%	1	0.12%	1	0.12
2-1-8: Elapsed timer	Normal	±0.01%	1	0.01%	1	0.01
2-2: Electrometer reading in numerator						
2-2-1: Repeatability	Normal	±0.1%	1	0.10%	1	0.1
2-2-2: Zero drift	Normal	±0.058%	1	0.058%	1	0.058
3: Correction for influence quantities						
3-1: Temperature-pressure correction in denominator						
3-1-1: Instrument error in thermometer	Normal	±0.3 ℃	1	0.3 ℃	0.34%/℃	0.10
3-1-2: Instrument error in barometer	Normal	±0.1 kPa	1	0.1 kPa	0.99%/kPa	0.10
3-2: Temperature-pressure correction in numerator						
3-2-1: Instrument error in thermometer	Normal	±0.3 ℃	1	0.3 ℃	0.34%/℃	0.10
3-2-2: Instrument error in barometer	Normal	±0.1 kPa	1	0.1 kPa	0.99%/kPa	0.10
Combined uncertainty	Normal					0.466
Expanded uncertainty (k = 2)						0.94

[a]Combining components 1-5-1, 1-5-2, and 1-5-3.

PDF, probability density function; SSD, source to surface distance

Supplementary Table 13. Realistic uncertainty in the measurement of the PDD in the inner region (at 10-cm depth) on the central axis of the 4 MV photon beam.

Component of uncertainty	PDF		Divisor	Standard uncertainty	Sensitivity coefficient	Uncertainty contribution (%)
	Shape	Variation limit/ Standard deviation/ Deflection				
1: Measurement conditions						
1-1: SSD setting	Uniform	±1 mm	1.73	0.58 mm	0.2%/mm	0.12
1-2: Setting chamber at depth of maximum dose	Normal	±0.24 mm[a]	1	0.24 mm	0.22%/mm	0.05
1-2-1: Setting origin in phantom	Uniform	±0.4 mm	1.73	0.23 mm	-	-
1-2-2: Reproducibility of movement distance	Uniform	±0.1 mm	1.73	0.06 mm	-	-
1-3: Setting chamber at 10-cm depth						
1-3-1: Reproducibility of movement distance	Uniform	±0.1 mm	1.73	0.06 mm	0.62%/mm	0.04
1-4: Field-size setting	Uniform	±1 mm	1.73	0.58 mm	0.13%/mm	0.07
1-5: Water evaporation	Normal	±0.62 mm[b]	1	0.62 mm	0.41%/mm	0.25
1-5-1: Water level adjustment among users	Uniform	±0.4 mm	1.73	0.23 mm	-	-
1-5-2: Mean displacement after water level adjustment	Deflection	0.5 mm	-	0.5 mm	-	-
1-5-3: Variation in water level	Uniform	±0.5 mm	1.73	0.29 mm	-	-
2: Charge measurement						
2-1: Electrometer reading in denominator						
2-1-1: Display resolution	Normal	±0.058%	1	0.058%	1	0.058
2-1-2: Repeatability	Normal	±0.1%	1	0.1%	1	0.1
2-1-3: Zero drift	Normal	±0.058%	1	0.058%	1	0.058
2-1-4: Non-linearity	Normal	±0.12%	1	0.12%	1	0.12
2-1-5: Response to pulsed beam from a linac	Normal	±0.12%	1	0.12%	1	0.12
2-1-6: Stabilization time	Normal	±0.12%	1	0.12%	1	0.12
2-1-7: Mains voltage fluctuation during measurement	Normal	±0.12%	1	0.12%	1	0.12
2-1-8: Elapsed timer	Normal	±0.01%	1	0.01%	1	0.01
2-2: Electrometer reading in numerator						
2-2-1: Repeatability	Normal	±0.1%	1	0.1%	1	0.1
2-2-2: Zero drift	Normal	±0.058%	1	0.058%	1	0.058
3: Correction for influence quantities						
3-1: Temperature-pressure correction in denominator						
3-1-1: Instrument error in thermometer	Uniform	±0.5 ℃	1.73	0.29 ℃	0.34%/℃	0.10
3-1-2: Instrument error in barometer	Uniform	±0.07 kPa	1.73	0.04 kPa	0.99%/kPa	0.04
3-2: Temperature-pressure correction in numerator						
3-2-1: Instrument error in thermometer	Uniform	±0.5 ℃	1.73	0.29 ℃	0.34%/℃	0.10
3-2-2: Instrument error in barometer	Uniform	±0.07 kPa	1.73	0.04 kPa	0.99%/kPa	0.04
Combined uncertainty	Normal					0.443
Expanded uncertainty (k = 2)						0.89

[a]Combining components 1-2-1 and 1-2-2.

[b]Combining components 1-5-1, 1-5-2, and 1-5-3.

PDF, probability density function; SSD, source to surface distance

Supplementary Table 14. Realistic uncertainty in the measurement of the PDD in the inner region (at 10-cm depth) on the central axis of the 6 MV photon beam.

Component of uncertainty	PDF		Divisor	Standard uncertainty	Sensitivity coefficient	Uncertainty contribution (%)
	Shape	Variation limit/ Standard deviation/ Deflection				
1: Measurement conditions						
1-1: SSD setting	Uniform	±1 mm	1.73	0.58 mm	0.2%/mm	0.12
1-2: Setting chamber at depth of maximum dose	Normal	±0.24 mm[a]	1	0.24 mm	0.06%/mm	0.02
1-2-1: Setting origin in phantom	Uniform	±0.4 mm	1.73	0.23 mm	-	-
1-2-2: Reproducibility of movement distance	Uniform	±0.1 mm	1.73	0.06 mm	-	-
1-3: Setting chamber at 10-cm depth						
1-3-1: Reproducibility of movement distance	Uniform	±0.1 mm	1.73	0.06 mm	0.55%/mm	0.03
1-4: Field-size setting	Uniform	±1 mm	1.73	0.58 mm	0.13%/mm	0.07
1-5: Water evaporation	Normal	±0.62 mm[b]	1	0.62 mm	0.39%/mm	0.24
1-5-1: Water level adjustment among users	Uniform	±0.4 mm	1.73	0.23 mm	-	-
1-5-2: Mean displacement after water level adjustment	Deflection	0.5 mm	-	0.5 mm	-	-
1-5-3: Variation in water level	Uniform	±0.5 mm	1.73	0.29 mm	-	-
2: Charge measurement						
2-1: Electrometer reading in denominator						
2-1-1: Display resolution	Normal	±0.058%	1	0.058%	1	0.058
2-1-2: Repeatability	Normal	±0.1%	1	0.1%	1	0.1
2-1-3: Zero drift	Normal	±0.058%	1	0.058%	1	0.058
2-1-4: Non-linearity	Normal	±0.12%	1	0.12%	1	0.12
2-1-5: Response to pulsed beam from a linac	Normal	±0.12%	1	0.12%	1	0.12
2-1-6: Stabilization time	Normal	±0.12%	1	0.12%	1	0.12
2-1-7: Mains voltage fluctuation during measurement	Normal	±0.12%	1	0.12%	1	0.12
2-1-8: Elapsed timer	Normal	±0.01%	1	0.01%	1	0.01
2-2: Electrometer reading in numerator						
2-2-1: Repeatability	Normal	±0.1%	1	0.1%	1	0.1
2-2-2: Zero drift	Normal	±0.058%	1	0.058%	1	0.058
3: Correction for influence quantities						
3-1: Temperature-pressure correction in denominator						
3-1-1: Instrument error in thermometer	Uniform	±0.5 ℃	1.73	0.29 ℃	0.34%/℃	0.10
3-1-2: Instrument error in barometer	Uniform	±0.07 kPa	1.73	0.04 kPa	0.99%/kPa	0.04
3-2: Temperature-pressure correction in numerator						
3-2-1: Instrument error in thermometer	Uniform	±0.5 ℃	1.73	0.29 ℃	0.34%/℃	0.10
3-2-2: Instrument error in barometer	Uniform	±0.07 kPa	1.73	0.04 kPa	0.99%/kPa	0.04
Combined uncertainty	Normal					0.433
Expanded uncertainty (k = 2)						0.87

[a]Combining components 1-2-1 and 1-2-2.

[b]Combining components 1-5-1, 1-5-2, and 1-5-3.

PDF, probability density function; SSD, source to surface distance

Supplementary Table 15. Realistic uncertainty in the measurement of the PDD in the inner region (at 10-cm depth) on the central axis of the 10 MV photon beam.

Component of uncertainty	PDF		Divisor	Standard uncertainty	Sensitivity coefficient	Uncertainty contribution (%)
	Shape	Variation limit/ Standard deviation/ Deflection				
1: Measurement conditions						
1-1: SSD setting	Uniform	±1 mm	1.73	0.58 mm	0.2%/mm	0.12
1-2: Setting chamber at depth of maximum dose	Normal	±0.24 mm[a]	1	0.24 mm	0.09%/mm	0.02
1-2-1: Setting origin in phantom	Uniform	±0.4 mm	1.73	0.23 mm	-	-
1-2-2: Reproducibility of movement distance	Uniform	±0.1 mm	1.73	0.06 mm	-	-
1-3: Setting chamber at 10-cm depth						
1-3-1: Reproducibility of movement distance	Uniform	±0.1 mm	1.73	0.06 mm	0.46%/mm	0.03
1-4: Field-size setting	Uniform	±1 mm	1.73	0.58 mm	0.10%/mm	0.06
1-5: Water evaporation	Normal	±0.62 mm[b]	1	0.62 mm	0.28%/mm	0.18
1-5-1: Water level adjustment among users	Uniform	±0.4 mm	1.73	0.23 mm	-	-
1-5-2: Mean displacement after water level adjustment	Deflection	0.5 mm	-	0.5 mm	-	-
1-5-3: Variation in water level	Uniform	±0.5 mm	1.73	0.29 mm	-	-
2: Charge measurement						
2-1: Electrometer reading in denominator						
2-1-1: Display resolution	Normal	±0.058%	1	0.058%	1	0.058
2-1-2: Repeatability	Normal	±0.1%	1	0.1%	1	0.1
2-1-3: Zero drift	Normal	±0.058%	1	0.058%	1	0.058
2-1-4: Non-linearity	Normal	±0.12%	1	0.12%	1	0.12
2-1-5: Response to pulsed beam from a linac	Normal	±0.12%	1	0.12%	1	0.12
2-1-6: Stabilization time	Normal	±0.12%	1	0.12%	1	0.12
2-1-7: Mains voltage fluctuation during measurement	Normal	±0.12%	1	0.12%	1	0.12
2-1-8: Elapsed timer	Normal	±0.01%	1	0.01%	1	0.01
2-2: Electrometer reading in numerator						
2-2-1: Repeatability	Normal	±0.1%	1	0.1%	1	0.1
2-2-2: Zero drift	Normal	±0.058%	1	0.058%	1	0.058
3: Correction for influence quantities						
3-1: Temperature-pressure correction in denominator						
3-1-1: Instrument error in thermometer	Uniform	±0.5 ℃	1.73	0.29 ℃	0.34%/℃	0.10
3-1-2: Instrument error in barometer	Uniform	±0.07 kPa	1.73	0.04 kPa	0.99%/kPa	0.04
3-2: Temperature-pressure correction in numerator						
3-2-1: Instrument error in thermometer	Uniform	±0.5 ℃	1.73	0.29 ℃	0.34%/℃	0.10
3-2-2: Instrument error in barometer	Uniform	±0.07 kPa	1.73	0.04 kPa	0.99%/kPa	0.04
Combined uncertainty	Normal					0.399
Expanded uncertainty (k = 2)						0.80

[a]Combining components 1-2-1 and 1-2-2.

[b]Combining components 1-5-1, 1-5-2, and 1-5-3.

PDF, probability density function; SSD, source to surface distance

Supplementary Table 16. Conservative uncertainty in the measurement of the PDD in the inner region (at 10-cm depth) on the central axis of the 4 MV photon beam.

Component of uncertainty	PDF		Divisor	Standard uncertainty	Sensitivity coefficient	Uncertainty contribution (%)
	Shape	Variation limit/ Standard deviation/ Deflection				
1: Measurement conditions						
1-1: SSD setting	Uniform	±2 mm	1.73	1.2 mm	0.2%/mm	0.23
1-2: Setting chamber at depth of maximum dose	Normal	±0.5 mm	1	0.5 mm	0.22%/mm	0.11
1-3: Setting chamber at 10-cm depth						
1-3-1: Accuracy of movement distance	Uniform	±0.1 mm	1.73	0.06 mm	0.62%/mm	0.04
1-4: Field-size setting	Uniform	±2 mm	1.73	1.2 mm	0.13%/mm	0.14
1-5: Water evaporation	Normal	±0.62 mm[a]	1	0.62 mm	0.41%/mm	0.25
1-5-1: Water level adjustment among users	Uniform	±0.4 mm	1.73	0.23 mm	-	
1-5-2: Mean displacement after water level adjustment	Deflection	0.5 mm	-	0.5 mm	-	
1-5-3: Variation in water level	Uniform	±0.5 mm	1.73	0.29 mm	-	
2: Charge measurement						
2-1: Electrometer reading in denominator						
2-1-1: Display resolution	Normal	±0.058%	1	0.058%	1	0.058
2-1-2: Repeatability	Normal	±0.1%	1	0.1%	1	0.1
2-1-3: Zero drift	Normal	±0.058%	1	0.058%	1	0.058
2-1-4: Non-linearity	Normal	±0.12%	1	0.12%	1	0.12
2-1-5: Response to pulsed beam from a linac	Normal	±0.12%	1	0.12%	1	0.12
2-1-6: Stabilization time	Normal	±0.12%	1	0.12%	1	0.12
2-1-7: Mains voltage fluctuation during measurement	Normal	±0.12%	1	0.12%	1	0.12
2-1-8: Elapsed timer	Normal	±0.01%	1	0.01%	1	0.01
2-2: Electrometer reading in numerator						
2-2-1: Repeatability	Normal	±0.1%	1	0.1%	1	0.1
2-2-2: Zero drift	Normal	±0.058%	1	0.058%	1	0.058
3: Correction for influence quantities						
3-1: Temperature-pressure correction in denominator						
3-1-1: Instrument error in thermometer	Normal	±0.3 ℃	1	0.3 ℃	0.34%/℃	0.10
3-1-2: Instrument error in barometer	Normal	±0.1 kPa	1	0.1 kPa	0.99%/kPa	0.10
3-2: Temperature-pressure correction in numerator						
3-2-1: Instrument error in thermometer	Normal	±0.3 ℃	1	0.3 ℃	0.34%/℃	0.10
3-2-2: Instrument error in barometer	Normal	±0.1 kPa	1	0.1 kPa	0.99%/kPa	0.10
Combined uncertainty	Normal					0.528
Expanded uncertainty (k = 2)						1.1

[a]Combining components 1-5-1, 1-5-2, and 1-5-3.

PDF, probability density function; SSD, source to surface distance

Supplementary Table 17. Conservative uncertainty in the measurement of the PDD in the inner region (at 10-cm depth) on the central axis of the 6 MV photon beam.

Component of uncertainty	PDF		Divisor	Standard uncertainty	Sensitivity coefficient	Uncertainty contribution (%)
	Shape	Variation limit/ Standard deviation/ Deflection				
1: Measurement conditions						
1-1: SSD setting	Uniform	±2 mm	1.73	1.2 mm	0.2%/mm	0.23
1-2: Setting chamber at depth of maximum dose	Normal	±0.5 mm	1	0.5 mm	0.06%/mm	0.03
1-3: Setting chamber at 10-cm depth						
1-3-1: Accuracy of movement distance	Uniform	±0.1 mm	1.73	0.06 mm	0.55%/mm	0.03
1-4: Field-size setting	Uniform	±2 mm	1.73	1.2 mm	0.13%/mm	0.14
1-5: Water evaporation	Normal	±0.62 mm[a]	1	0.62 mm	0.39%/mm	0.24
1-5-1: Water level adjustment among users	Uniform	±0.4 mm	1.73	0.23 mm	-	
1-5-2: Mean displacement after water level adjustment	Deflection	0.5 mm	-	0.5 mm	-	
1-5-3: Variation in water level	Uniform	±0.5 mm	1.73	0.29 mm	-	
2: Charge measurement						
2-1: Electrometer reading in denominator						
2-1-1: Display resolution	Normal	±0.058%	1	0.058%	1	0.058
2-1-2: Repeatability	Normal	±0.1%	1	0.1%	1	0.1
2-1-3: Zero drift	Normal	±0.058%	1	0.058%	1	0.058
2-1-4: Non-linearity	Normal	±0.12%	1	0.12%	1	0.12
2-1-5: Response to pulsed beam from a linac	Normal	±0.12%	1	0.12%	1	0.12
2-1-6: Stabilization time	Normal	±0.12%	1	0.12%	1	0.12
2-1-7: Mains voltage fluctuation during measurement	Normal	±0.12%	1	0.12%	1	0.12
2-1-8: Elapsed timer	Normal	±0.01%	1	0.01%	1	0.01
2-2: Electrometer reading in numerator						
2-2-1: Repeatability	Normal	±0.1%	1	0.1%	1	0.1
2-2-2: Zero drift	Normal	±0.058%	1	0.058%	1	0.058
3: Correction for influence quantities						
3-1: Temperature-pressure correction in denominator						
3-1-1: Instrument error in thermometer	Normal	±0.3 ℃	1	0.3 ℃	0.34%/℃	0.10
3-1-2: Instrument error in barometer	Normal	±0.1 kPa	1	0.1 kPa	0.99%/kPa	0.10
3-2: Temperature-pressure correction in numerator						
3-2-1: Instrument error in thermometer	Normal	±0.3 ℃	1	0.3 ℃	0.34%/℃	0.10
3-2-2: Instrument error in barometer	Normal	±0.1 kPa	1	0.1 kPa	0.99%/kPa	0.10
Combined uncertainty	Normal					0.512
Expanded uncertainty (k = 2)						1.1

[a]Combining components 1-5-1, 1-5-2, and 1-5-3.

PDF, probability density function; SSD, source to surface distance

Supplementary Table 18. Conservative uncertainty in the measurement of the PDD in the inner region (at 10-cm depth) on the central axis of the 10 MV photon beam.

Component of uncertainty	PDF		Divisor	Standard uncertainty	Sensitivity coefficient	Uncertainty contribution (%)
	Shape	Variation limit/ Standard deviation/ Deflection				
1: Measurement conditions						
1-1: SSD setting	Uniform	±2 mm	1.73	1.2 mm	0.2%/mm	0.23
1-2: Setting chamber at depth of maximum dose	Normal	±0.5 mm	1	0.5 mm	0.09%/mm	0.04
1-3: Setting chamber at 10-cm depth						
1-3-1: Accuracy of movement distance	Uniform	±0.1 mm	1.73	0.06 mm	0.46%/mm	0.03
1-4: Field-size setting	Uniform	±2 mm	1.73	1.2 mm	0.1%/mm	0.12
1-5: Water evaporation	Normal	±0.62 mm[a]	1	0.62 mm	0.28%/mm	0.18
1-5-1: Water level adjustment among users	Uniform	±0.4 mm	1.73	0.23 mm	-	
1-5-2: Mean displacement after water level adjustment	Deflection	0.5 mm	-	0.5 mm	-	
1-5-3: Variation in water level	Uniform	±0.5 mm	1.73	0.29 mm	-	
2: Charge measurement						
2-1: Electrometer reading in denominator						
2-1-1: Display resolution	Normal	±0.058%	1	0.058%	1	0.058
2-1-2: Repeatability	Normal	±0.1%	1	0.1%	1	0.1
2-1-3: Zero drift	Normal	±0.058%	1	0.058%	1	0.058
2-1-4: Non-linearity	Normal	±0.12%	1	0.12%	1	0.12
2-1-5: Response to pulsed beam from a linac	Normal	±0.12%	1	0.12%	1	0.12
2-1-6: Stabilization time	Normal	±0.12%	1	0.12%	1	0.12
2-1-7: Mains voltage fluctuation during measurement	Normal	±0.12%	1	0.12%	1	0.12
2-1-8: Elapsed timer	Normal	±0.01%	1	0.01%	1	0.01
2-2: Electrometer reading in numerator						
2-2-1: Repeatability	Normal	±0.1%	1	0.1%	1	0.1
2-2-2: Zero drift	Normal	±0.058%	1	0.058%	1	0.058
3: Correction for influence quantities						
3-1: Temperature-pressure correction in denominator						
3-1-1: Instrument error in thermometer	Normal	±0.3 ℃	1	0.3 ℃	0.34%/℃	0.10
3-1-2: Instrument error in barometer	Normal	±0.1 kPa	1	0.1 kPa	0.99%/kPa	0.10
3-2: Temperature-pressure correction in numerator						
3-2-1: Instrument error in thermometer	Normal	±0.3 ℃	1	0.3 ℃	0.34%/℃	0.10
3-2-2: Instrument error in barometer	Normal	±0.1 kPa	1	0.1 kPa	0.99%/kPa	0.10
Combined uncertainty	Normal					0.478
Expanded uncertainty (k = 2)						0.96

[a]Combining components 1-5-1, 1-5-2, and 1-5-3.

PDF, probability density function; SSD, source to surface distance

Supplementary Table 19. Realistic uncertainty in the OCR measurement at 4 cm off-axis from the central axis of the 4 MV photon beam at 10-cm depth.

Component of uncertainty	PDF		Divisor	Standard uncertainty	Sensitivity coefficient	Uncertainty contribution (%)
	Shape	Variation limit/ Standard deviation/ Deflection				
1: Measurement conditions						
1-1: SSD setting	Uniform	±1 mm	1.73	0.58 mm	0.2%/mm	0.12
1-2: Setting chamber on central beam axis at 10-cm depth	Normal	±0.24 mm[a]	1	0.24 mm	0.62%/mm	0.15
1-2-1: Setting origin in phantom	Uniform	±0.4 mm	1.73	0.23 mm	-	
1-2-2: Reproducibility of movement distance	Uniform	±0.1 mm	1.73	0.06 mm	-	
1-3: Setting chamber on 4 cm off-central axis						
1-3-1: Reproducibility of movement distance	Uniform	±0.1 mm	1.73	0.06 mm	0.23%/mm	0.01
1-4: Field-size setting						
1-4-1: Dosimeter reading on central beam axis at 10 cm	Uniform	±1 mm	1.73	0.58 mm	0.13%/mm	0.07
1-4-2: Dosimeter reading at 4 cm off-central axis	Uniform	±1 mm	1.73	0.58 mm	0.29%/mm	0.17
1-5: Water evaporation	Normal	±0.62 mm[b]	1	0.62 mm	0.41%/mm	0.25
1-5-1: Water level adjustment among users	Uniform	±0.4 mm	1.73	0.23 mm	-	
1-5-2: Mean displacement from water level adjustment	Deflection	0.5 mm	-	0.5 mm	-	
1-5-3: Variation in water level	Uniform	±0.5 mm	1.73	0.29 mm	-	
2: Charge measurement						
2-1: Electrometer reading in denominator						
2-1-1: Display resolution	Normal	±0.058%	1	0.058%	1	0.058
2-1-2: Repeatability	Normal	±0.1%	1	0.1%	1	0.1
2-1-3: Zero drift	Normal	±0.058%	1	0.058%	1	0.058
2-1-4: Non-linearity	Normal	±0.12%	1	0.12%	1	0.12
2-1-5: Response to pulsed beam from a linac	Normal	±0.12%	1	0.12%	1	0.12
2-1-6: Stabilization time	Normal	±0.12%	1	0.12%	1	0.12
2-1-7: Mains voltage fluctuation during measurement	Normal	±0.12%	1	0.12%	1	0.12
2-1-8: Elapsed timer	Normal	±0.01%	1	0.01%	1	0.01
2-2: Electrometer reading in numerator						
2-2-1: Repeatability	Normal	±0.1%	1	0.1%	1	0.1
2-2-2: Zero drift	Normal	±0.058%	1	0.058%	1	0.058
3: Correction for influence quantities						
3-1: Temperature-pressure correction in denominator						
3-1-1: Instrument error in thermometer	Uniform	±0.5 ℃	1.73	0.29 ℃	0.34%/℃	0.10
3-1-2: Instrument error in barometer	Uniform	±0.07 kPa	1.73	0.04 kPa	0.99%/kPa	0.04
3-2: Temperature-pressure correction in numerator						
3-2-1: Instrument error in thermometer	Uniform	±0.5 ℃	1.73	0.29 ℃	0.34%/℃	0.10
3-2-2: Instrument error in barometer	Uniform	±0.07 kPa	1.73	0.04 kPa	0.99%/kPa	0.04
Combined uncertainty	Normal					0.492
Expanded uncertainty (k = 2)						0.99

[a]Combining components 1-2-1 and 1-2-2.

[b]Combining components 1-5-1, 1-5-2, and 1-5-3.

PDF, probability density function; SSD, source to surface distance

Supplementary Table 20. Realistic uncertainty in the OCR measurement at 4 cm off-axis from the central axis of the 6 MV photon beam at 10-cm depth.

Component of uncertainty	PDF		Divisor	Standard uncertainty	Sensitivity coefficient	Uncertainty contribution (%)
	Shape	Variation limit/ Standard deviation/ Deflection				
1: Measurement conditions						
1-1: SSD setting	Uniform	±1 mm	1.73	0.58 mm	0.2%/mm	0.12
1-2: Setting chamber on central beam axis at 10-cm depth	Normal	±0.24 mm[a]	1	0.24 mm	0.55%/mm	0.13
1-2-1: Setting origin in phantom	Uniform	±0.4 mm	1.73	0.23 mm	-	
1-2-2: Reproducibility of movement distance	Uniform	±0.1 mm	1.73	0.06 mm	-	
1-3: Setting chamber on 4 cm off-central axis						
1-3-1: Reproducibility of movement distance	Uniform	±0.1 mm	1.73	0.06 mm	0.23%/mm	0.01
1-4: Field-size setting						
1-4-1: Dosimeter reading on central beam axis at 10 cm	Uniform	±1 mm	1.73	0.58 mm	0.13%/mm	0.07
1-4-2: Dosimeter reading at 4 cm off-central axis	Uniform	±1 mm	1.73	0.58 mm	0.25%/mm	0.15
1-5: Water evaporation	Normal	±0.62 mm[b]	1	0.62 mm	0.39%/mm	0.24
1-5-1: Water level adjustment among users	Uniform	±0.4 mm	1.73	0.23 mm	-	
1-5-2: Mean displacement from water level adjustment	Deflection	0.5 mm	-	0.5 mm	-	
1-5-3: Variation in water level	Uniform	±0.5 mm	1.73	0.29 mm	-	
2: Charge measurement						
2-1: Electrometer reading in denominator						
2-1-1: Display resolution	Normal	±0.058%	1	0.058%	1	0.058
2-1-2: Repeatability	Normal	±0.1%	1	0.1%	1	0.1
2-1-3: Zero drift	Normal	±0.058%	1	0.058%	1	0.058
2-1-4: Non-linearity	Normal	±0.12%	1	0.12%	1	0.12
2-1-5: Response to pulsed beam from a linac	Normal	±0.12%	1	0.12%	1	0.12
2-1-6: Stabilization time	Normal	±0.12%	1	0.12%	1	0.12
2-1-7: Mains voltage fluctuation during measurement	Normal	±0.12%	1	0.12%	1	0.12
2-1-8: Elapsed timer	Normal	±0.01%	1	0.01%	1	0.01
2-2: Electrometer reading in numerator						
2-2-1: Repeatability	Normal	±0.1%	1	0.1%	1	0.1
2-2-2: Zero drift	Normal	±0.058%	1	0.058%	1	0.058
3: Correction for influence quantities						
3-1: Temperature-pressure correction in denominator						
3-1-1: Instrument error in thermometer	Uniform	±0.5 ℃	1.73	0.29 ℃	0.34%/℃	0.10
3-1-2: Instrument error in barometer	Uniform	±0.07 kPa	1.73	0.04 kPa	0.99%/kPa	0.04
3-2: Temperature-pressure correction in numerator						
3-2-1: Instrument error in thermometer	Uniform	±0.5 ℃	1.73	0.29 ℃	0.34%/℃	0.10
3-2-2: Instrument error in barometer	Uniform	±0.07 kPa	1.73	0.04 kPa	0.99%/kPa	0.04
Combined uncertainty	Normal					0.474
Expanded uncertainty (k = 2)						0.95

[a]Combining components 1-2-1 and 1-2-2.

[b]Combining components 1-5-1, 1-5-2, and 1-5-3.

PDF, probability density function; SSD, source to surface distance

Supplementary Table 21. Realistic uncertainty in the OCR measurement at 4 cm off-axis from the central axis of the 10 MV photon beam at 10-cm depth.

Component of uncertainty	PDF		Divisor	Standard uncertainty	Sensitivity coefficient	Uncertainty contribution (%)
	Shape	Variation limit/ Standard deviation/ Deflection				
1: Measurement conditions						
1-1: SSD setting	Uniform	±1 mm	1.73	0.58 mm	0.2%/mm	0.12
1-2: Setting chamber on central beam axis at 10-cm depth	Normal	±0.24 mm[a]	1	0.24 mm	0.46%/mm	0.11
1-2-1: Setting origin in phantom	Uniform	±0.4 mm	1.73	0.23 mm	-	
1-2-2: Reproducibility of movement distance	Uniform	±0.1 mm	1.73	0.06 mm	-	
1-3: Setting chamber on 4 cm off-central axis						
1-3-1: Reproducibility of movement distance	Uniform	±0.1 mm	1.73	0.06 mm	0.25%/mm	0.01
1-4: Field-size setting						
1-4-1: Dosimeter reading on central beam axis at 10 cm	Uniform	±1 mm	1.73	0.58 mm	0.10%/mm	0.06
1-4-2: Dosimeter reading at 4 cm off-central axis	Uniform	±1 mm	1.73	0.58 mm	0.24%/mm	0.14
1-5: Water evaporation	Normal	±0.62 mm[b]	1	0.62 mm	0.28%/mm	0.18
1-5-1: Water level adjustment among users	Uniform	±0.4 mm	1.73	0.23 mm	-	
1-5-2: Mean displacement from water level adjustment	Deflection	0.5 mm	-	0.5 mm	-	
1-5-3: Variation in water level	Uniform	±0.5 mm	1.73	0.29 mm	-	
2: Charge measurement						
2-1: Electrometer reading in denominator						
2-1-1: Display resolution	Normal	±0.058%	1	0.058%	1	0.058
2-1-2: Repeatability	Normal	±0.1%	1	0.1%	1	0.1
2-1-3: Zero drift	Normal	±0.058%	1	0.058%	1	0.058
2-1-4: Non-linearity	Normal	±0.12%	1	0.12%	1	0.12
2-1-5: Response to pulsed beam from a linac	Normal	±0.12%	1	0.12%	1	0.12
2-1-6: Stabilization time	Normal	±0.12%	1	0.12%	1	0.12
2-1-7: Mains voltage fluctuation during measurement	Normal	±0.12%	1	0.12%	1	0.12
2-1-8: Elapsed timer	Normal	±0.01%	1	0.01%	1	0.01
2-2: Electrometer reading in numerator						
2-2-1: Repeatability	Normal	±0.1%	1	0.1%	1	0.1
2-2-2: Zero drift	Normal	±0.058%	1	0.058%	1	0.058
3: Correction for influence quantities						
3-1: Temperature-pressure correction in denominator						
3-1-1: Instrument error in thermometer	Uniform	±0.5 ℃	1.73	0.29 ℃	0.34%/℃	0.10
3-1-2: Instrument error in barometer	Uniform	±0.07 kPa	1.73	0.04 kPa	0.99%/kPa	0.04
3-2: Temperature-pressure correction in numerator						
3-2-1: Instrument error in thermometer	Uniform	±0.5 ℃	1.73	0.29 ℃	0.34%/℃	0.10
3-2-2: Instrument error in barometer	Uniform	±0.07 kPa	1.73	0.04 kPa	0.99%/kPa	0.04
Combined uncertainty	Normal					0.435
Expanded uncertainty (*k* = 2)						0.87

[a]Combining components 1-2-1 and 1-2-2.

[b]Combining components 1-5-1, 1-5-2, and 1-5-3.

PDF, probability density function; SSD, source to surface distance

Supplementary Table 22. Conservative uncertainty in the OCR measurement at 4 cm off-axis from the central axis of the 4 MV photon beam at 10-cm depth.

Component of uncertainty	PDF		Divisor	Standard uncertainty	Sensitivity coefficient	Uncertainty contribution (%)
	Shape	Variation limit/ Standard deviation/ Deflection				
1: Measurement conditions						
1-1: SSD setting	Uniform	±2 mm	1.73	1.2 mm	0.2%/mm	0.23
1-2: Setting chamber on central beam axis at 10-cm depth	Normal	±0.5 mm	1	0.5 mm	0.62 %/mm	0.31
1-3: Setting chamber at 4 cm off-central axis						
1-3-1: Accuracy of movement distance	Uniform	±0.1 mm	1.73	0.06 mm	0.23%/mm	0.01
1-4: Field-size setting						
1-4-1: Dosimeter reading on central beam axis at 10 cm	Uniform	±2 mm	1.73	1.2 mm	0.13%/mm	0.14
1-4-2: Dosimeter reading at 4 cm off-central axis	Uniform	±2 mm	1.73	1.2 mm	0.29%/mm	0.33
1-5: Water evaporation	Normal	±0.62 mm[a]	1	0.62 mm	0.41%/mm	0.25
1-5-1: Water level adjustment among users	Uniform	±0.4 mm	1.73	0.23 mm	-	
1-5-2: Mean displacement from water level adjustment	Deflection	0.5 mm	-	0.5 mm	-	
1-5-3: Variation in water level	Uniform	±0.5 mm	1.73	0.29 mm	-	
2: Charge measurement						
2-1: Electrometer reading in denominator						
2-1-1: Display resolution	Normal	±0.058%	1	0.058%	1	0.058
2-1-2: Repeatability	Normal	±0.1%	1	0.1%	1	0.1
2-1-3: Zero drift	Normal	±0.058%	1	0.058%	1	0.058
2-1-4: Non-linearity	Normal	±0.12%	1	0.12%	1	0.12
2-1-5: Response to pulsed beam from a linac	Normal	±0.12%	1	0.12%	1	0.12
2-1-6: Stabilization time	Normal	±0.12%	1	0.12%	1	0.12
2-1-7: Mains voltage fluctuation during measurement	Normal	±0.12%	1	0.12%	1	0.12
2-1-8: Elapsed timer	Normal	±0.01%	1	0.01%	1	0.01
2-2: Electrometer reading in numerator						
2-2-1: Repeatability	Normal	±0.1%	1	0.1%	1	0.1
2-2-2: Zero drift	Normal	±0.058%	1	0.058%	1	0.058
3: Correction for influence quantities						
3-1: Temperature-pressure correction in denominator						
3-1-1: Instrument error in thermometer	Normal	±0.3 ℃	1	0.3 ℃	0.34%/℃	0.10
3-1-2: Instrument error in barometer	Normal	±0.1 kPa	1	0.1 kPa	0.99%/kPa	0.10
3-2: Temperature-pressure correction in numerator						
3-2-1: Instrument error in thermometer	Normal	±0.3 ℃	1	0.3 ℃	0.34%/℃	0.10
3-2-2: Instrument error in barometer	Normal	±0.1 kPa	1	0.1 kPa	0.99%/kPa	0.10
Combined uncertainty	Normal					0.687
Expanded uncertainty (k = 2)						1.4

[a]Combining components 1-5-1, 1-5-2, and 1-5-3.

PDF, probability density function; SSD, source to surface distance

Supplementary Table 23. Conservative uncertainty in the OCR measurement at 4 cm off-axis from the central axis of the 6 MV photon beam at 10-cm depth.

Component of uncertainty	PDF		Divisor	Standard uncertainty	Sensitivity coefficient	Uncertainty contribution (%)
	Shape	Variation limit/ Standard deviation/ Deflection				
1: Measurement conditions						
1-1: SSD setting	Uniform	±2 mm	1.73	1.2 mm	0.2%/mm	0.23
1-2: Setting chamber on central beam axis at 10-cm depth	Normal	±0.5 mm	1	0.5 mm	0.55 %/mm	0.27
1-3: Setting chamber at 4 cm off-central axis						
1-3-1: Accuracy of movement distance	Uniform	±0.1 mm	1.73	0.06 mm	0.23%/mm	0.01
1-4: Field-size setting						
1-4-1: Dosimeter reading on central beam axis at 10 cm	Uniform	±2 mm	1.73	1.2 mm	0.13%/mm	0.14
1-4-2: Dosimeter reading at 4 cm off-central axis	Uniform	±2 mm	1.73	1.2 mm	0.25%/mm	0.29
1-5: Water evaporation	Normal	±0.62 mm[a]	1	0.62 mm	0.39%/mm	0.24
1-5-1: Water level adjustment among users	Uniform	±0.4 mm	1.73	0.23 mm	-	
1-5-2: Mean displacement from water level adjustment	Deflection	0.5 mm	-	0.5 mm	-	
1-5-3: Variation in water level	Uniform	±0.5 mm	1.73	0.29 mm	-	
2: Charge measurement						
2-1: Electrometer reading in denominator						
2-1-1: Display resolution	Normal	±0.058%	1	0.058%	1	0.058
2-1-2: Repeatability	Normal	±0.1%	1	0.1%	1	0.1
2-1-3: Zero drift	Normal	±0.058%	1	0.058%	1	0.058
2-1-4: Non-linearity	Normal	±0.12%	1	0.12%	1	0.12
2-1-5: Response to pulsed beam from a linac	Normal	±0.12%	1	0.12%	1	0.12
2-1-6: Stabilization time	Normal	±0.12%	1	0.12%	1	0.12
2-1-7: Mains voltage fluctuation during measurement	Normal	±0.12%	1	0.12%	1	0.12
2-1-8: Elapsed timer	Normal	±0.01%	1	0.01%	1	0.01
2-2: Electrometer reading in numerator						
2-2-1: Repeatability	Normal	±0.1%	1	0.1%	1	0.1
2-2-2: Zero drift	Normal	±0.058%	1	0.058%	1	0.058
3: Correction for influence quantities						
3-1: Temperature-pressure correction in denominator						
3-1-1: Instrument error in thermometer	Normal	±0.3 ℃	1	0.3 ℃	0.34%/℃	0.10
3-1-2: Instrument error in barometer	Normal	±0.1 kPa	1	0.1 kPa	0.99%/kPa	0.10
3-2: Temperature-pressure correction in numerator						
3-2-1: Instrument error in thermometer	Normal	±0.3 ℃	1	0.3 ℃	0.34%/℃	0.10
3-2-2: Instrument error in barometer	Normal	±0.1 kPa	1	0.1 kPa	0.99%/kPa	0.10
Combined uncertainty	Normal					0.648
Expanded uncertainty (k = 2)						1.3

[a]Combining components 1-5-1, 1-5-2, and 1-5-3.

PDF, probability density function; SSD, source to surface distance

Supplementary Table 24. Conservative uncertainty in the OCR measurement at 4 cm off-axis from the central axis of the 10 MV photon beam at 10-cm depth.

Component of uncertainty	PDF		Divisor	Standard uncertainty	Sensitivity coefficient	Uncertainty contribution (%)
	Shape	Variation limit/ Standard deviation/ Deflection				
1: Measurement conditions						
1-1: SSD setting	Uniform	±2 mm	1.73	1.2 mm	0.2%/mm	0.23
1-2: Setting chamber on central beam axis at 10-cm depth	Normal	±0.5 mm	1	0.5 mm	0.46 %/mm	0.23
1-3: Setting chamber at 4 cm off-central axis						
1-3-1: Accuracy of movement distance	Uniform	±0.1 mm	1.73	0.06 mm	0.25%/mm	0.01
1-4: Field-size setting						
1-4-1: Dosimeter reading on central beam axis at 10 cm	Uniform	±2 mm	1.73	1.2 mm	0.10%/mm	0.12
1-4-2: Dosimeter reading at 4 cm off-central axis	Uniform	±2 mm	1.73	1.2 mm	0.24%/mm	0.27
1-5: Water evaporation	Normal	±0.62 mm[a]	1	0.62 mm	0.28%/mm	0.18
1-5-1: Water level adjustment among users	Uniform	±0.4 mm	1.73	0.23 mm	-	
1-5-2: Mean displacement from water level adjustment	Deflection	0.5 mm	-	0.5 mm	-	
1-5-3: Variation in water level	Uniform	±0.5 mm	1.73	0.29 mm	-	
2: Charge measurement						
2-1: Electrometer reading in denominator						
2-1-1: Display resolution	Normal	±0.058%	1	0.058%	1	0.058
2-1-2: Repeatability	Normal	±0.1%	1	0.1%	1	0.1
2-1-3: Zero drift	Normal	±0.058%	1	0.058%	1	0.058
2-1-4: Non-linearity	Normal	±0.12%	1	0.12%	1	0.12
2-1-5: Response to pulsed beam from a linac	Normal	±0.12%	1	0.12%	1	0.12
2-1-6: Stabilization time	Normal	±0.12%	1	0.12%	1	0.12
2-1-7: Mains voltage fluctuation during measurement	Normal	±0.12%	1	0.12%	1	0.12
2-1-8: Elapsed timer	Normal	±0.01%	1	0.01%	1	0.01
2-2: Electrometer reading in numerator						
2-2-1: Repeatability	Normal	±0.1%	1	0.1%	1	0.1
2-2-2: Zero drift	Normal	±0.058%	1	0.058%	1	0.058
3: Correction for influence quantities						
3-1: Temperature-pressure correction in denominator						
3-1-1: Instrument error in thermometer	Normal	±0.3 ℃	1	0.3 ℃	0.34%/℃	0.10
3-1-2: Instrument error in barometer	Normal	±0.1 kPa	1	0.1 kPa	0.99%/kPa	0.10
3-2: Temperature-pressure correction in numerator						
3-2-1: Instrument error in thermometer	Normal	±0.3 ℃	1	0.3 ℃	0.34%/℃	0.10
3-2-2: Instrument error in barometer	Normal	±0.1 kPa	1	0.1 kPa	0.99%/kPa	0.10
Combined uncertainty	Normal					0.595
Expanded uncertainty (k = 2)						1.2

[a]Combining components 1-5-1, 1-5-2, and 1-5-3.

PDF, probability density function; SSD, source to surface distance

Supplementary Table 25. Realistic uncertainty in the OCR measurement at 5.5 cm off-axis from the central axis of the 4 MV photon beam at 10-cm depth.

Component of uncertainty	PDF		Divisor	Standard uncertainty	Sensitivity coefficient	Uncertainty contribution (%)
	Shape	Variation limit/ Standard deviation/ Deflection				
1: Measurement conditions						
1-1: SSD setting	Uniform	±1 mm	1.73	0.58 mm	0.2%/mm	0.12
1-2: Setting chamber on central beam axis at 10-cm depth	Normal	±0.24 mm[a]	1	0.24 mm	0.62%/mm	0.15
1-2-1: Setting origin in phantom	Uniform	±0.4 mm	1.73	0.23 mm	-	
1-2-2: Reproducibility of movement distance	Uniform	±0.1 mm	1.73	0.06 mm	-	
1-3: Setting chamber at 5.5 cm off-central axis						
1-3-1: Reproducibility of movement distance	Uniform	±0.1 mm	1.73	0.06 mm	23.7%/mm	1.4
1-4: Field-size setting						
1-4-1: Dosimeter reading on central beam axis at 10 cm	Uniform	±1 mm	1.73	0.58 mm	0.13%/mm	0.07
1-4-2: Dosimeter reading at 5.5 cm off-central axis	Uniform	±1 mm	1.73	0.58 mm	12.3%/mm	7.1
1-5: Water evaporation	Normal	±0.62 mm[b]	1	0.62 mm	0.41%/mm	0.25
1-5-1: Water level adjustment among users	Uniform	±0.4 mm	1.73	0.23 mm	-	
1-5-2: Mean displacement from water level adjustment	Deflection	0.5 mm	-	0.5 mm	-	
1-5-3: Variation in water level	Uniform	±0.5 mm	1.73	0.29 mm	-	
2: Charge measurement						
2-1: Electrometer reading in denominator						
2-1-1: Display resolution	Normal	±0.058%	1	0.058%	1	0.058
2-1-2: Repeatability	Normal	±0.1%	1	0.1%	1	0.1
2-1-3: Zero drift	Normal	±0.058%	1	0.058%	1	0.058
2-1-4: Non-linearity	Normal	±0.12%	1	0.12%	1	0.12
2-1-5: Response to pulsed beam from a linac	Normal	±0.12%	1	0.12%	1	0.12
2-1-6: Stabilization time	Normal	±0.12%	1	0.12%	1	0.12
2-1-7: Mains voltage fluctuation during measurement	Normal	±0.12%	1	0.12%	1	0.12
2-1-8: Elapsed timer	Normal	±0.01%	1	0.01%	1	0.01
2-2: Electrometer reading in numerator						
2-2-1: Repeatability	Normal	±0.1%	1	0.1%	1	0.1
2-2-2: Zero drift	Normal	±0.058%	1	0.058%	1	0.058
3: Correction for influence quantities						
3-1: Temperature-pressure correction in denominator						
3-1-1: Instrument error in thermometer	Uniform	±0.5 ℃	1.73	0.29 ℃	0.34%/℃	0.10
3-1-2: Instrument error in barometer	Uniform	±0.07 kPa	1.73	0.04 kPa	0.99%/kPa	0.04
3-2: Temperature-pressure correction in numerator						
3-2-1: Instrument error in thermometer	Uniform	±0.5 ℃	1.73	0.29 ℃	0.34%/℃	0.10
3-2-2: Instrument error in barometer	Uniform	±0.07 kPa	1.73	0.04 kPa	0.99%/kPa	0.04
Combined uncertainty	Normal					7.23
Expanded uncertainty (k = 2)						15

[a]Combining components 1-2-1 and 1-2-2.

[b]Combining components 1-5-1, 1-5-2, and 1-5-3.

PDF, probability density function; SSD, source to surface distance

Supplementary Table 26. Realistic uncertainty in the OCR measurement at 5.5 cm off-axis from the central axis of the 6 MV photon beam at 10-cm depth.

Component of uncertainty	PDF		Divisor	Standard uncertainty	Sensitivity coefficient	Uncertainty contribution (%)
	Shape	Variation limit/ Standard deviation/ Deflection				
1: Measurement conditions						
1-1: SSD setting	Uniform	±1 mm	1.73	0.58 mm	0.2%/mm	0.12
1-2: Setting chamber on central beam axis at 10-cm depth	Normal	±0.24 mm[a]	1	0.24 mm	0.55%/mm	0.13
1-2-1: Setting origin in phantom	Uniform	±0.4 mm	1.73	0.23 mm	-	
1-2-2: Reproducibility of movement distance	Uniform	±0.1 mm	1.73	0.06 mm	-	
1-3: Setting chamber at 5.5 cm off-central axis						
1-3-1: Reproducibility of movement distance	Uniform	±0.1 mm	1.73	0.06 mm	23.5%/mm	1.4
1-4: Field-size setting						
1-4-1: Dosimeter reading on central beam axis at 10 cm	Uniform	±1 mm	1.73	0.58 mm	0.13%/mm	0.07
1-4-2: Dosimeter reading at 5.5 cm off-central axis	Uniform	±1 mm	1.73	0.58 mm	12.1%/mm	7.0
1-5: Water evaporation	Normal	±0.62 mm[b]	1	0.62 mm	0.39%/mm	0.24
1-5-1: Water level adjustment among users	Uniform	±0.4 mm	1.73	0.23 mm	-	
1-5-2: Mean displacement from water level adjustment	Deflection	0.5 mm	-	0.5 mm	-	
1-5-3: Variation in water level	Uniform	±0.5 mm	1.73	0.29 mm	-	
2: Charge measurement						
2-1: Electrometer reading in denominator						
2-1-1: Display resolution	Normal	±0.058%	1	0.058%	1	0.058
2-1-2: Repeatability	Normal	±0.1%	1	0.1%	1	0.1
2-1-3: Zero drift	Normal	±0.058%	1	0.058%	1	0.058
2-1-4: Non-linearity	Normal	±0.12%	1	0.12%	1	0.12
2-1-5: Response to pulsed beam from a linac	Normal	±0.12%	1	0.12%	1	0.12
2-1-6: Stabilization time	Normal	±0.12%	1	0.12%	1	0.12
2-1-7: Mains voltage fluctuation during measurement	Normal	±0.12%	1	0.12%	1	0.12
2-1-8: Elapsed timer	Normal	±0.01%	1	0.01%	1	0.01
2-2: Electrometer reading in numerator						
2-2-1: Repeatability	Normal	±0.1%	1	0.1%	1	0.1
2-2-2: Zero drift	Normal	±0.058%	1	0.058%	1	0.058
3: Correction for influence quantities						
3-1: Temperature-pressure correction in denominator						
3-1-1: Instrument error in thermometer	Uniform	±0.5 ℃	1.73	0.29 ℃	0.34%/℃	0.10
3-1-2: Instrument error in barometer	Uniform	±0.07 kPa	1.73	0.04 kPa	0.99%/kPa	0.04
3-2: Temperature-pressure correction in numerator						
3-2-1: Instrument error in thermometer	Uniform	±0.5 ℃	1.73	0.29 ℃	0.34%/℃	0.10
3-2-2: Instrument error in barometer	Uniform	±0.07 kPa	1.73	0.04 kPa	0.99%/kPa	0.04
Combined uncertainty	Normal					7.11
Expanded uncertainty (k = 2)						15

[a]Combining components 1-2-1 and 1-2-2.

[b]Combining components 1-5-1, 1-5-2, and 1-5-3.

PDF, probability density function; SSD, source to surface distance

Supplementary Table 27. Realistic uncertainty in the OCR measurement at 5.5 cm off-axis from the central axis of the 10 MV photon beam at 10-cm depth.

Component of uncertainty	PDF		Divisor	Standard uncertainty	Sensitivity coefficient	Uncertainty contribution (%)
	Shape	Variation limit/ Standard deviation/ Deflection				
1: Measurement conditions						
1-1: SSD setting	Uniform	±1 mm	1.73	0.58 mm	0.2%/mm	0.12
1-2: Setting chamber on central beam axis at 10-cm depth	Normal	±0.24 mm[a]	1	0.24 mm	0.46%/mm	0.11
1-2-1: Setting origin in phantom	Uniform	±0.4 mm	1.73	0.23 mm	-	
1-2-2: Reproducibility of movement distance	Uniform	±0.1 mm	1.73	0.06 mm	-	
1-3: Setting chamber at 5.5 cm off-central axis						
1-3-1: Reproducibility of movement distance	Uniform	±0.1 mm	1.73	0.06 mm	21.7%/mm	1.3
1-4: Field-size setting						
1-4-1: Dosimeter reading on central beam axis at 10 cm	Uniform	±1 mm	1.73	0.58 mm	0.10%/mm	0.06
1-4-2: Dosimeter reading at 5.5 cm off-central axis	Uniform	±1 mm	1.73	0.58 mm	10.9%/mm	6.3
1-5: Water evaporation	Normal	±0.62 mm[b]	1	0.62 mm	0.28%/mm	0.18
1-5-1: Water level adjustment among users	Uniform	±0.4 mm	1.73	0.23 mm	-	
1-5-2: Mean displacement from water level adjustment	Deflection	0.5 mm	-	0.5 mm	-	
1-5-3: Variation in water level	Uniform	±0.5 mm	1.73	0.29 mm	-	
2: Charge measurement						
2-1: Electrometer reading in denominator						
2-1-1: Display resolution	Normal	±0.058%	1	0.058%	1	0.058
2-1-2: Repeatability	Normal	±0.1%	1	0.1%	1	0.1
2-1-3: Zero drift	Normal	±0.058%	1	0.058%	1	0.058
2-1-4: Non-linearity	Normal	±0.12%	1	0.12%	1	0.12
2-1-5: Response to pulsed beam from a linac	Normal	±0.12%	1	0.12%	1	0.12
2-1-6: Stabilization time	Normal	±0.12%	1	0.12%	1	0.12
2-1-7: Mains voltage fluctuation during measurement	Normal	±0.12%	1	0.12%	1	0.12
2-1-8: Elapsed timer	Normal	±0.01%	1	0.01%	1	0.01
2-2: Electrometer reading in numerator						
2-2-1: Repeatability	Normal	±0.1%	1	0.1%	1	0.1
2-2-2: Zero drift	Normal	±0.058%	1	0.058%	1	0.058
3: Correction for influence quantities						
3-1: Temperature-pressure correction in denominator						
3-1-1: Instrument error in thermometer	Uniform	±0.5 ℃	1.73	0.29 ℃	0.34%/℃	0.10
3-1-2: Instrument error in barometer	Uniform	±0.07 kPa	1.73	0.04 kPa	0.99%/kPa	0.04
3-2: Temperature-pressure correction in numerator						
3-2-1: Instrument error in thermometer	Uniform	±0.5 ℃	1.73	0.29 ℃	0.34%/℃	0.10
3-2-2: Instrument error in barometer	Uniform	±0.07 kPa	1.73	0.04 kPa	0.99%/kPa	0.04
Combined uncertainty	Normal					6.44
Expanded uncertainty (k = 2)						13

[a]Combining components 1-2-1 and 1-2-2.

[b]Combining components 1-5-1, 1-5-2, and 1-5-3.

PDF, probability density function; SSD, source to surface distance

Supplementary Table 28. Conservative uncertainty in the OCR measurement at 5.5 cm off-axis from the central axis of the 4 MV photon beam at 10-cm depth.

Component of uncertainty	PDF		Divisor	Standard uncertainty	Sensitivity coefficient	Uncertainty contribution (%)
	Shape	Variation limit/ Standard deviation/ Deflection				
1: Measurement conditions						
1-1: SSD setting	Uniform	±2 mm	1.73	1.2 mm	0.2%/mm	0.23
1-2: Setting chamber on central beam axis at 10-cm depth	Normal	±0.5 mm	1	0.5 mm	0.62%/mm	0.31
1-3: Setting chamber at 5.5 cm off-central axis						
1-3-1: Accuracy of movement distance	Uniform	±0.1 mm	1.73	0.06 mm	23.7%/mm	1.4
1-4: Field-size setting						
1-4-1: Dosimeter reading on central beam axis at 10 cm	Uniform	±2 mm	1.73	1.2 mm	0.13%/mm	0.14
1-4-2: Dosimeter reading at 5.5 cm off-central axis	Uniform	±2 mm	1.73	1.2 mm	12.3%/mm	14.2
1-5: Water evaporation	Normal	±0.62 mm[a]	1	0.62 mm[a]	0.41%/mm	0.25
1-5-1: Water level adjustment among users	Uniform	±0.4 mm	1.73	0.23 mm	-	
1-5-2: Mean displacement from water level adjustment	Deflection	0.5 mm	-	0.5 mm	-	
1-5-3: Variation in water level	Uniform	±0.5 mm	1.73	0.29 mm	-	
2: Charge measurement						
2-1: Electrometer reading in denominator						
2-1-1: Display resolution	Normal	±0.058%	1	0.058%	1	0.058
2-1-2: Repeatability	Normal	±0.1%	1	0.1%	1	0.1
2-1-3: Zero drift	Normal	±0.058%	1	0.058%	1	0.058
2-1-4: Non-linearity	Normal	±0.12%	1	0.12%	1	0.12
2-1-5: Response to pulsed beam from a linac	Normal	±0.12%	1	0.12%	1	0.12
2-1-6: Stabilization time	Normal	±0.12%	1	0.12%	1	0.12
2-1-7: Mains voltage fluctuation during measurement	Normal	±0.12%	1	0.12%	1	0.12
2-1-8: Elapsed timer	Normal	±0.01%	1	0.01%	1	0.01
2-2: Electrometer reading in numerator						
2-2-1: Repeatability	Normal	±0.1%	1	0.1%	1	0.1
2-2-2: Zero drift	Normal	±0.058%	1	0.058%	1	0.058
3: Correction for influence quantities						
3-1: Temperature-pressure correction in denominator						
3-1-1: Instrument error in thermometer	Normal	±0.3 ℃	1	0.3 ℃	0.34%/℃	0.10
3-1-2: Instrument error in barometer	Normal	±0.1 kPa	1	0.1 kPa	0.99%/kPa	0.10
3-2: Temperature-pressure correction in numerator						
3-2-1: Instrument error in thermometer	Normal	±0.3 ℃	1	0.3 ℃	0.34%/℃	0.10
3-2-2: Instrument error in barometer	Normal	±0.1 kPa	1	0.1 kPa	0.99%/kPa	0.10
Combined uncertainty	Normal					14.2
Expanded uncertainty (k = 2)						29

[a]Combining components 1-5-1, 1-5-2, and 1-5-3.

PDF, probability density function; SSD, source to surface distance

Supplementary Table 29. Conservative uncertainty in the OCR measurement at 5.5 cm off-axis from the central axis of the 6 MV photon beam at 10-cm depth.

Component of uncertainty	PDF		Divisor	Standard uncertainty	Sensitivity coefficient	Uncertainty contribution (%)
	Shape	Variation limit/ Standard deviation/ Deflection				
1: Measurement conditions						
1-1: SSD setting	Uniform	±2 mm	1.73	1.2 mm	0.2%/mm	0.23
1-2: Setting chamber on central beam axis at 10-cm depth	Normal	±0.5 mm	1	0.5 mm	0.55 %/mm	0.27
1-3: Setting chamber at 5.5 cm off-central axis						
1-3-1: Accuracy of movement distance	Uniform	±0.1 mm	1.73	0.06 mm	23.5%/mm	1.4
1-4: Field-size setting						
1-4-1: Dosimeter reading on central beam axis at 10 cm	Uniform	±2 mm	1.73	1.2 mm	0.13%/mm	0.14
1-4-2: Dosimeter reading at 5.5 cm off-central axis	Uniform	±2 mm	1.73	1.2 mm	12.1%/mm	13.9
1-5: Water evaporation	Normal	±0.62 mm[a]	1	0.62 mm	0.39%/mm	0.24
1-5-1: Water level adjustment among users	Uniform	±0.4 mm	1.73	0.23 mm	-	
1-5-2: Mean displacement from water level adjustment	Deflection	0.5 mm	-	0.5 mm	-	
1-5-3: Variation in water level	Uniform	±0.5 mm	1.73	0.29 mm	-	
2: Charge measurement						
2-1: Electrometer reading in denominator						
2-1-1: Display resolution	Normal	±0.058%	1	0.058%	1	0.058
2-1-2: Repeatability	Normal	±0.1%	1	0.1%	1	0.1
2-1-3: Zero drift	Normal	±0.058%	1	0.058%	1	0.058
2-1-4: Non-linearity	Normal	±0.12%	1	0.12%	1	0.12
2-1-5: Response to pulsed beam from a linac	Normal	±0.12%	1	0.12%	1	0.12
2-1-6: Stabilization time	Normal	±0.12%	1	0.12%	1	0.12
2-1-7: Mains voltage fluctuation during measurement	Normal	±0.12%	1	0.12%	1	0.12
2-1-8: Elapsed timer	Normal	±0.01%	1	0.01%	1	0.01
2-2: Electrometer reading in numerator						
2-2-1: Repeatability	Normal	±0.1%	1	0.1%	1	0.1
2-2-2: Zero drift	Normal	±0.058%	1	0.058%	1	0.058
3: Correction for influence quantities						
3-1: Temperature-pressure correction in denominator						
3-1-1: Instrument error in thermometer	Normal	±0.3 °C	1	0.3 °C	0.34%/°C	0.10
3-1-2: Instrument error in barometer	Normal	±0.1 kPa	1	0.1 kPa	0.99%/kPa	0.10
3-2: Temperature-pressure correction in numerator						
3-2-1: Instrument error in thermometer	Normal	±0.3 °C	1	0.3 °C	0.34%/°C	0.10
3-2-2: Instrument error in barometer	Normal	±0.1 kPa	1	0.1 kPa	0.99%/kPa	0.10
Combined uncertainty	Normal					14.0
Expanded uncertainty (k = 2)						28

[a]Combining components 1-5-1, 1-5-2, and 1-5-3.

PDF, probability density function; SSD, source to surface distance

Supplementary Table 30. Conservative uncertainty in the OCR measurement at 5.5 cm off-axis from the central axis of the 10 MV photon beam at 10-cm depth.

Component of uncertainty	PDF		Divisor	Standard uncertainty	Sensitivity coefficient	Uncertainty contribution (%)
	Shape	Variation limit/ Standard deviation/ Deflection				
1: Measurement conditions						
1-1: SSD setting	Uniform	±2 mm	1.73	1.2 mm	0.2%/mm	0.23
1-2: Setting chamber on central beam axis at 10-cm depth	Normal	±0.5 mm	1	0.5 mm	0.46%/mm	0.23
1-3: Setting chamber at 5.5 cm off-central axis						
1-3-1: Accuracy of movement distance	Uniform	±0.1 mm	1.73	0.06 mm	21.7%/mm	1.3
1-4: Field-size setting						
1-4-1: Dosimeter reading on central beam axis at 10 cm	Uniform	±2 mm	1.73	1.2 mm	0.10%/mm	0.12
1-4-2: Dosimeter reading at 5.5 cm off-central axis	Uniform	±2 mm	1.73	1.2 mm	10.9%/mm	12.6
1-5: Water evaporation	Normal	±0.62 mm[a]	1	0.62 mm	0.28%/mm	0.18
1-5-1: Water level adjustment among users	Uniform	±0.4 mm	1.73	0.23 mm	-	
1-5-2: Mean displacement from water level adjustment	Deflection	0.5 mm	-	0.5 mm	-	
1-5-3: Variation in water level	Uniform	±0.5 mm	1.73	0.29 mm	-	
2: Charge measurement						
2-1: Electrometer reading in denominator						
2-1-1: Display resolution	Normal	±0.058%	1	0.058%	1	0.058
2-1-2: Repeatability	Normal	±0.1%	1	0.1%	1	0.1
2-1-3: Zero drift	Normal	±0.058%	1	0.058%	1	0.058
2-1-4: Non-linearity	Normal	±0.12%	1	0.12%	1	0.12
2-1-5: Response to pulsed beam from a linac	Normal	±0.12%	1	0.12%	1	0.12
2-1-6: Stabilization time	Normal	±0.12%	1	0.12%	1	0.12
2-1-7: Mains voltage fluctuation during measurement	Normal	±0.12%	1	0.12%	1	0.12
2-1-8: Elapsed timer	Normal	±0.01%	1	0.01%	1	0.01
2-2: Electrometer reading in numerator						
2-2-1: Repeatability	Normal	±0.1%	1	0.1%	1	0.1
2-2-2: Zero drift	Normal	±0.058%	1	0.058%	1	0.058
3: Correction for influence quantities						
3-1: Temperature-pressure correction in denominator						
3-1-1: Instrument error in thermometer	Normal	±0.3 ℃	1	0.3 ℃	0.34%/℃	0.10
3-1-2: Instrument error in barometer	Normal	±0.1 kPa	1	0.1 kPa	0.99%/kPa	0.10
3-2: Temperature-pressure correction in numerator						
3-2-1: Instrument error in thermometer	Normal	±0.3 ℃	1	0.3 ℃	0.34%/℃	0.10
3-2-2: Instrument error in barometer	Normal	±0.1 kPa	1	0.1 kPa	0.99%/kPa	0.10
Combined uncertainty	Normal					12.7
Expanded uncertainty (k = 2)						26

[a]Combining components 1-5-1, 1-5-2, and 1-5-3.

PDF, probability density function; SSD, source to surface distance

Supplementary Table 31. Realistic uncertainty in PDD calculation for the 4 MV photon beam in the buildup region.

Component of uncertainty	PDF		Divisor	Standard uncertainty	Sensitivity coefficient	Uncertainty contribution (%)
	Shape	Variation limit				
1: CT number	Uniform	±0.5 HU	1.73	0.29 HU	0.05%/HU	0.01
2: PDD in the region of buildup	Uniform	±1.7%	1.73	0.98%	1	0.98
3: Beam attenuation by a treatment couch	Uniform	±0.44%	1.73	0.25%	1	0.25
Combined standard uncertainty	Normal					1.01
Expanded uncertainty (*k* = 2)						2.1

PDF, probability density function; CT, computed tomography; HU, Hounsfield unit

Supplementary Table 32. Realistic uncertainty in PDD calculation for the 6 MV photon beam in the buildup region.

Component of uncertainty	PDF		Divisor	Standard uncertainty	Sensitivity coefficient	Uncertainty contribution (%)
	Shape	Variation limit				
1: CT number	Uniform	±0.5 HU	1.73	0.29 HU	0.05%/HU	0.01
2: PDD in the region of buildup	Uniform	±0.90%	1.73	0.52%	1	0.52
3: Beam attenuation by a treatment couch	Uniform	±0.32%	1.73	0.18%	1	0.18
Combined standard uncertainty	Normal					0.552
Expanded uncertainty (*k* = 2)						1.1

PDF, probability density function; CT, computed tomography; HU, Hounsfield unit

Supplementary Table 33. Realistic uncertainty in PDD calculation for the 10 MV photon beam in the buildup region.

Component of uncertainty	PDF		Divisor	Standard uncertainty	Sensitivity coefficient	Uncertainty contribution (%)
	Shape	Variation limit				
1: CT number	Uniform	±0.5 HU	1.73	0.29 HU	0.05%/HU	0.01
2: PDD in the region of buildup	Uniform	±2.4%	1.73	1.4%	1	1.4
3: Beam attenuation by a treatment couch	Uniform	±0.24%	1.73	0.14%	1	0.14
Combined standard uncertainty	Normal					1.39
Expanded uncertainty (*k* = 2)						2.8

PDF, probability density function; CT, computed tomography; HU, Hounsfield unit

Supplementary Table 34. Conservative uncertainty in PDD calculations for the 4, 6, and 10 MV photon beams in the buildup regions.

Component of uncertainty	PDF		Divisor	Standard uncertainty	Sensitivity coefficient	Uncertainty contribution (%)
	Shape	Variation limit				
1: CT number	Uniform	±5 HU	1.73	2.9 HU	0.05 %/HU	0.14
2: PDD in the region of buildup	Uniform	±10%	1.73	5.8%	1	5.8
3: Beam attenuation by a treatment couch	Uniform	±0.6%	1.73	0.35%	1	0.35
Combined standard uncertainty	Normal					5.79
Expanded uncertainty (*k* = 2)						12

PDF, probability density function; CT, computed tomography; HU, Hounsfield unit

Supplementary Table 35. Realistic uncertainty in PDD calculation for the 4 MV photon beam in the inner region.

Component of uncertainty	PDF		Divisor	Standard uncertainty	Sensitivity coefficient	Uncertainty contribution (%)
	Shape	Variation limit				
1: CT number	Uniform	±0.5 HU	1.73	0.29 HU	0.05 %/HU	0.01
2: PDD in the region of inner	Uniform	±1.1%	1.73	0.64%	1	0.64
3: Beam attenuation by a treatment couch	Uniform	±0.44%	1.73	0.25%	1	0.25
Combined standard uncertainty	Normal					0.684
Expanded uncertainty (*k* = 2)						1.4

PDF, probability density function; CT, computed tomography; HU, Hounsfield unit

Supplementary Table 36. Realistic uncertainty in PDD calculation for the 6 MV photon beam in the inner region.

Component of uncertainty	PDF		Divisor	Standard uncertainty	Sensitivity coefficient	Uncertainty contribution (%)
	Shape	Variation limit				
1: CT number	Uniform	±0.5 HU	1.73	0.29 HU	0.05 %/HU	0.01
2: PDD in the region of inner	Uniform	±0.5%	1.73	0.29%	1	0.29
3: Beam attenuation by a treatment couch	Uniform	±0.32%	1.73	0.18%	1	0.18
Combined standard uncertainty	Normal					0.343
Expanded uncertainty (*k* = 2)						0.69

PDF, probability density function; CT, computed tomography; HU, Hounsfield unit

Supplementary Table 37. Realistic uncertainty in PDD calculation for the 10 MV photon beam in the inner region.

Component of uncertainty	PDF		Divisor	Standard uncertainty	Sensitivity coefficient	Uncertainty contribution (%)
	Shape	Variation limit				
1: CT number	Uniform	±0.5 HU	1.73	0.29 HU	0.05 %/HU	0.01
2: PDD in the region of inner	Uniform	±1.2%	1.73	0.69%	1	0.69
3: Beam attenuation by a treatment couch	Uniform	±0.24%	1.73	0.14%	1	0.14
Combined standard uncertainty	Normal					0.707
Expanded uncertainty (*k* = 2)						1.5

PDF, probability density function; CT, computed tomography; HU, Hounsfield unit

Supplementary Table 38. Conservative uncertainty in PDD calculation for the 4, 6, and 10 MV photon beams in the inner regions.

Component of uncertainty	PDF		Divisor	Standard uncertainty	Sensitivity coefficient	Uncertainty contribution (%)
	Shape	Variation limit				
1: CT number	Uniform	±5 HU	1.73	2.9 HU	0.05 %/HU	0.14
2: PDD in the region of inner	Uniform	±2%	1.73	1.2%	1	1.2
3: Beam attenuation by a treatment couch	Uniform	±0.6%	1.73	0.35%	1	0.35
Combined standard uncertainty	Normal					1.21
Expanded uncertainty (*k* = 2)						2.5

PDF, probability density function; CT, computed tomography; HU, Hounsfield unit

Supplementary Table 39. Realistic uncertainty in OCR calculation for the 4 MV photon beam in the inner region.

Component of uncertainty	PDF		Divisor	Standard uncertainty	Sensitivity coefficient	Uncertainty contribution (%)
	Shape	Variation limit				
1: CT number	Uniform	±0.5 HU	1.73	0.29 HU	0.05 %/HU	0.01
2: OCR in the region of inner	Uniform	±3.4%	1.73	2.0%	1	2.0
3: Beam attenuation by a treatment couch	Uniform	±0.44%	1.73	0.25%	1	0.25
Combined standard uncertainty	Normal					1.98
Expanded uncertainty (*k* = 2)						4.0

PDF, probability density function; CT, computed tomography; HU, Hounsfield unit

Supplementary Table 40. Realistic uncertainty in OCR calculation for the 6 MV photon beam in the inner region.

Component of uncertainty	PDF		Divisor	Standard uncertainty	Sensitivity coefficient	Uncertainty contribution (%)
	Shape	Variation limit				
1: CT number	Uniform	±0.5 HU	1.73	0.29 HU	0.05 %/HU	0.01
2: OCR in the region of inner	Uniform	±3.0%	1.73	1.7%	1	1.7
3: Beam attenuation by a treatment couch	Uniform	±0.32%	1.73	0.18%	1	0.18
Combined standard uncertainty	Normal					1.74
Expanded uncertainty (*k* = 2)						3.5

PDF, probability density function; CT, computed tomography; HU, Hounsfield unit

Supplementary Table 41. Realistic uncertainty in OCR calculation for the 10 MV photon beam in the inner region.

Component of uncertainty	PDF		Divisor	Standard uncertainty	Sensitivity coefficient	Uncertainty contribution (%)
	Shape	Variation limit				
1: CT number	Uniform	±0.5 HU	1.73	0.29 HU	0.05 %/HU	0.01
2: OCR in the region of inner	Uniform	±2.4%	1.73	1.4%	1	1.4
3: Beam attenuation by a treatment couch	Uniform	±0.24%	1.73	0.14%	1	0.14
Combined standard uncertainty	Normal					1.39
Expanded uncertainty (*k* = 2)						2.8

PDF, probability density function; CT, computed tomography; HU, Hounsfield unit

Supplementary Table 42. Conservative uncertainty in OCR calculations for the 4, 6, and 10 MV photon beams in the inner regions.

Component of uncertainty	PDF		Divisor	Standard uncertainty	Sensitivity coefficient	Uncertainty contribution (%)
	Shape	Variation limit				
1: CT number	Uniform	±5 HU	1.73	2.9 HU	0.05 %/HU	0.14
2: OCR in the region of inner	Uniform	±3%	1.73	1.7%	1	1.7
3: Beam attenuation by a treatment couch	Uniform	±0.6%	1.73	0.35%	1	0.35
Combined standard uncertainty	Normal					1.77
Expanded uncertainty (*k* = 2)						3.6

PDF, probability density function; CT, computed tomography; HU, Hounsfield unit

Supplementary Table 43. Realistic uncertainty in OCR calculation for the 4 MV photon beam in the penumbra region.

Component of uncertainty	PDF		Divisor	Standard uncertainty	Sensitivity coefficient	Uncertainty contribution (%)
	Shape	Variation limit				
1: CT number	Uniform	±0.5 HU	1.73	0.29 HU	0.05 %/HU	0.01
2: OCR in the region of penumbra	Uniform	±26.4%	1.73	15.2%	1	15.2
3: Beam attenuation by a treatment couch	Uniform	±0.44%	1.73	0.25%	1	0.25
Combined standard uncertainty	Normal					15.2
Expanded uncertainty (k = 2)						31

PDF, probability density function; CT, computed tomography; HU, Hounsfield unit

Supplementary Table 44. Realistic uncertainty in OCR calculation for the 6 MV photon beam in the penumbra region.

Component of uncertainty	PDF		Divisor	Standard uncertainty	Sensitivity coefficient	Uncertainty contribution (%)
	Shape	Variation limit				
1: CT number	Uniform	±0.5 HU	1.73	0.29 HU	0.05 %/HU	0.01
2: OCR in the region of penumbra	Uniform	±26.0%	1.73	15.0%	1	15.0
3: Beam attenuation by a treatment couch	Uniform	±0.32%	1.73	0.18%	1	0.18
Combined standard uncertainty	Normal					15.0
Expanded uncertainty (k = 2)						30

PDF, probability density function; CT, computed tomography; HU, Hounsfield unit

Supplementary Table 45. Realistic uncertainty in OCR calculation for the 10 MV photon beam in the penumbra region.

Component of uncertainty	PDF		Divisor	Standard uncertainty	Sensitivity coefficient	Uncertainty contribution (%)
	Shape	Variation limit				
1: CT number	Uniform	±0.5 HU	1.73	0.29 HU	0.05 %/HU	0.01
2: OCR in the region of penumbra	Uniform	±30.4%	1.73	17.6%	1	17.6
3: Beam attenuation by a treatment couch	Uniform	±0.24%	1.73	0.14%	1	0.14
Combined standard uncertainty	Normal					17.6
Expanded uncertainty (k = 2)						36

PDF, probability density function; CT, computed tomography; HU, Hounsfield unit

Supplementary Table 46. Conservative uncertainty in OCR calculations for the 4, 6, and 10 MV photon beams in the penumbra regions.

Component of uncertainty	PDF		Divisor	Standard uncertainty	Sensitivity coefficient	Uncertainty contribution (%)
	Shape	Variation limit				
1: CT number	Uniform	±5 HU	1.73	2.9 HU	0.05 %/HU	0.14
2: OCR in the region of penumbra	Uniform	±10%	1.73	5.8%	1	5.8
3: Beam attenuation by a treatment couch	Uniform	±0.6%	1.73	0.35%	1	0.35
Combined standard uncertainty	Normal					5.79
Expanded uncertainty (k = 2)						12

PDF, probability density function; CT, computed tomography; HU, Hounsfield unit

Supplementary Table 47. Realistic uncertainty in patient positioning with the IGRT system.

Component of uncertainty	PDF		Divisor	Standard uncertainty	Sensitivity coefficient	Uncertainty contribution (mm)
	Shape	Standard deviation				
1: Image registration	Normal	±0.22 mm	1	0.22	1	0.22
2: Coincidence of imaging isocenter and radiation isocenter	Normal	±0.30 mm	1	0.30	1	0.30
3: Variation in radiation isocenter due to gantry rotation	Normal	±0.23 mm	1	0.23	1	0.23
4: Variation in radiation isocenter due to couch rotation	Normal	±0.22 mm	1	0.22	1	0.22
Combined standard uncertainty	Normal					0.490
Expanded uncertainty (k = 2)						0.98

PDF, probability density function

Supplementary Table 48. Conservative uncertainty in patient positioning with the IGRT system.

Component of uncertainty	PDF		Divisor	Standard uncertainty	Sensitivity coefficient	Uncertainty contribution (mm)
	Shape	Variation limit				
1: Image registration	Uniform	±2 mm	1.73	1.2	1	1.2
2: Coincidence of imaging isocenter and radiation isocenter	Uniform	±2 mm	1.73	1.2	1	1.2
3: Variation in radiation isocenter due to gantry rotation	Uniform	±1 mm	1.73	0.58	1	0.58
4: Variation in radiation isocenter due to couch rotation	Uniform	±1 mm	1.73	0.58	1	0.58
Combined standard uncertainty	Normal					1.83
Expanded uncertainty (k = 2)						3.7

PDF, probability density function

Supplementary Table 49. Realistic uncertainty in intrafractional head motion under conventionally fractionated irradiation.

Component of uncertainty	PDF		Divisor	Standard uncertainty	Sensitivity coefficient	Uncertainty contribution (mm)
	Shape	Standard deviation/ Deflection				
1: Mean displacement from imaging isocenter	Deflection	1.06 mm	-	1.06	1	1.06
2: Variation in intrafractional motion	Normal	±1.21 mm	1	1.21	1	1.21
Combined standard uncertainty	Normal					1.61
Expanded uncertainty (k = 2)						3.3

PDF, probability density function

Supplementary Table 50. Conservative uncertainty in intrafractional head motion under conventionally fractionated irradiation.

Component of uncertainty	PDF		Divisor	Standard uncertainty	Sensitivity coefficient	Uncertainty contribution (mm)
	Shape	Variation limit				
1: Variation in intrafractional motion	Uniform	±3 mm	1.73	1.73	1	1.73
Combined standard uncertainty	Normal					1.73
Expanded uncertainty (k = 2)						3.5

PDF, probability density function

Supplementary Table 51. Realistic uncertainty in the reference dosimetry of the 4 MV photon beam using the ionization chamber calibrated in MV photon beams.

Component of uncertainty	PDF		Divisor	Standard uncertainty	Sensitivity coefficient	Uncertainty contribution (%)
	Shape	Variation limit/ Standard deviation				
1: Reference conditions						
1-1: SCD setting	Uniform	±1 mm[a]	1.73	0.58 mm	0.2%/mm	0.12
1-2: Chamber setting	Normal	±0.26 mm[b]	1	0.26 mm	0.66%/mm	0.18
1-2-1: Setting origin in phantom	Uniform	±0.4 mm[a]	1.73	0.23 mm	-	
1-2-2: Position accuracy	Uniform	±0.2 mm[a]	1.73	0.12 mm	-	
1-2-3: Position reproducibility	Uniform	±0.1 mm[a]	1.73	0.06 mm	-	
1-3: Field-size setting	Uniform	±1 mm[a]	1.73	0.58 mm	0.20%/mm	0.11
2: Charge measurement	Normal	±0.35%[c]	1	0.35%	1	0.35
2-1: Electrometer	Normal	±0.35%[a]	1	0.35%	-	
2-2: M_{raw} relative to 100 MU	Normal	±0.01%[a]	1	0.01%	-	
3: Long-term stability of ion chamber	Uniform	±0.2%[a]	1.73	0.12%	1	0.12
4: Correction for influence quantities						
4-1: Pressure and temperature						
4-1-1: Instrument error in thermometer	Uniform	±0.5 °C[a]	1.73	0.29 °C	0.34%/°C	0.10
4-1-2: Instrument error in barometer	Uniform	±0.07 kPa[a]	1.73	0.04 kPa	0.99%/kPa	0.04
4-2: Polarity effect						
4-2-1: $\frac{M_+}{M_-}$	Normal	±0.02%[a]	1	0.02%	0.5	0.01
4-3: Ion Recombination						
4-3-1: $\frac{M_1}{M_2}$	Normal	±0.02%[a]	1	0.02%	0.48	0.01
4-4: Humidity	Normal	±0.15%[a]	1	0.15%	1	0.15
5: Calibration of dosimeter						
5-1: Ion chamber[d]	Normal	±0.5%[e]	1	0.5%	1	0.5
5-2: Electrometer	Normal	±0.15%[a]	2	0.075%	1	0.075
6: Determination of $N_{\text{D,w,Q}}$ at the 4 MV photon beam[f]	Normal	±0.3%[e]	1	0.3%	1	0.3
Combined standard uncertainty	Normal					0.757
Expanded uncertainty (k = 2)						1.6

[a]Derived from the variation limit or standard deviation in the realistic estimate of Table 1.

[b]Combining components 1-2-1, 1-2-2, and 1-2-3.

[c]Combining components 2-1 and 2-2.

[d]Calibrated directly in MV photon beams.

[e]Quoted from Ref. [45].

[f]Uncertainty in the determination of a calibration coefficient of absorbed dose to water ($N_{\text{D,w,Q}}$) at a user beam quality Q using a polynomial approximation [45].

PDF, probability density function; SCD, source-chamber distance; M_{raw}, mean chamber reading; MU, monitor units; M_+ and M_-, mean-chamber readings obtained at polarizing voltages of −300 and +300 V, respectively; M_1 and M_2, mean-collected charges obtained at polarizing voltages of +300 and +100 V, respectively; $N_{\text{D,w,Q}}$, calibration coefficient of absorbed dose to water at a user beam quality Q

Supplementary Table 52. Realistic uncertainty in the reference dosimetry of the 6 MV photon beam using the ionization chamber calibrated in MV photon beams.

Component of uncertainty	PDF		Divisor	Standard uncertainty	Sensitivity coefficient	Uncertainty contribution (%)
	Shape	Variation limit/ Standard deviation				
1: Reference conditions						
1-1: SCD setting	Uniform	±1 mm[a]	1.73	0.58 mm	0.2%/mm	0.12
1-2: Chamber setting	Normal	±0.26 mm[b]	1	0.26 mm	0.57%/mm	0.15
1-2-1: Setting origin in phantom	Uniform	±0.4 mm[a]	1.73	0.23 mm	-	
1-2-2: Position accuracy	Uniform	±0.2 mm[a]	1.73	0.12 mm	-	
1-2-3: Position reproducibility	Uniform	±0.1 mm[a]	1.73	0.06 mm	-	
1-3: Field-size setting	Uniform	±1 mm[a]	1.73	0.58 mm	0.15%/mm	0.09
2: Charge measurement	Normal	±0.35%[c]	1	0.35%	1	0.35
2-1: Electrometer	Normal	±0.35%[a]	1	0.35%	-	
2-2: M_{raw} relative to 100 MU	Normal	±0.01%[a]	1	0.01%	-	
3: Long-term stability of ion chamber	Uniform	±0.2%[a]	1.73	0.12%	1	0.12
4: Correction for influence quantities						
4-1: Pressure and temperature						
4-1-1: Instrument error in thermometer	Uniform	±0.5 °C[a]	1.73	0.29 °C	0.34%/°C	0.10
4-1-2: Instrument error in barometer	Uniform	±0.07 kPa[a]	1.73	0.04 kPa	0.99%/kPa	0.04
4-2: Polarity effect						
4-2-1: $\frac{M_+}{M_-}$	Normal	±0.01%[a]	1	0.01%	0.5	0.006
4-3: Ion Recombination						
4-3-1: $\frac{M_1}{M_2}$	Normal	±0.01%[a]	1	0.01%	0.48	0.005
4-4: Humidity	Normal	±0.15%[a]	1	0.15%	1	0.15
5: Calibration of dosimeter						
5-1: Ion chamber[d]	Normal	±0.5%[e]	1	0.5%	1	0.5
5-2: Electrometer	Normal	±0.15%[a]	2	0.075%	1	0.075
6: Determination of $N_{\mathrm{D,w,Q}}$ at the 6 MV photon beam[f]	Normal	±0.3%[e]	1	0.3%	1	0.3
Combined standard uncertainty	Normal					0.747
Expanded uncertainty (k = 2)						1.5

[a]Derived from the variation limit or standard deviation in the realistic estimate of Table 1.

[b]Combining components 1-2-1, 1-2-2, and 1-2-3.

[c]Combining components 2-1 and 2-2.

[d]Calibrated directly in MV photon beams.

[e]Quoted from Ref. [45].

[f]Uncertainty in the determination of a calibration coefficient of absorbed dose to water ($N_{\mathrm{D,w,Q}}$) at a user beam quality Q using a polynomial approximation [45].

PDF, probability density function; SCD, source-chamber distance; M_{raw}, mean chamber reading; MU, monitor units; M_+ and M_-, mean-chamber readings obtained at polarizing voltages of −300 and +300 V, respectively; M_1 and M_2, mean-collected charges obtained at polarizing voltages of +300 and +100 V, respectively; $N_{\mathrm{D,w,Q}}$, calibration coefficient of absorbed dose to water at a user beam quality Q

Supplementary Table 53. Realistic uncertainty in the reference dosimetry of the 10 MV photon beam using the ionization chamber calibrated in MV photon beams.

Component of uncertainty	PDF		Divisor	Standard uncertainty	Sensitivity coefficient	Uncertainty contribution (%)
	Shape	Variation limit/ Standard deviation				
1: Reference conditions						
1-1: SCD setting	Uniform	±1 mm[a]	1.73	0.58 mm	0.2%/mm	0.12
1-2: Chamber setting	Normal	±0.26 mm[b]	1	0.26 mm	0.47%/mm	0.13
1-2-1: Setting origin in phantom	Uniform	±0.4 mm[a]	1.73	0.23 mm	-	
1-2-2: Position accuracy	Uniform	±0.2 mm[a]	1.73	0.12 mm	-	
1-2-3: Position reproducibility	Uniform	±0.1 mm[a]	1.73	0.06 mm	-	
1-3: Field-size setting	Uniform	±1 mm[a]	1.73	0.58 mm	0.11%/mm	0.07
2: Charge measurement	Normal	±0.35%[c]	1	0.35%	1	0.35
2-1: Electrometer	Normal	±0.35%[a]	1	0.35%	-	
2-2: M_{raw} relative to 100 MU	Normal	±0.005%[a]	1	0.005%	-	
3: Long-term stability of ion chamber	Uniform	±0.2%[a]	1.73	0.12%	1	0.12
4: Correction for influence quantities						
4-1: Pressure and temperature						
4-1-1: Instrument error in thermometer	Uniform	±0.5 °C[a]	1.73	0.29 °C	0.34%/°C	0.10
4-1-2: Instrument error in barometer	Uniform	±0.07 kPa[a]	1.73	0.04 kPa	0.99%/kPa	0.04
4-2: Polarity effect						
4-2-1: $\frac{M_+}{M_-}$	Normal	±0.005%[a]	1	0.005%	0.5	0.003
4-3: Ion Recombination						
4-3-1: $\frac{M_1}{M_2}$	Normal	±0.008%[a]	1	0.008%	0.48	0.004
4-4: Humidity	Normal	±0.15%[a]	1	0.15%	1	0.15
5: Calibration of dosimeter						
5-1: Ion chamber[d]	Normal	±0.5%[e]	1	0.5%	1	0.5
5-2: Electrometer	Normal	±0.15%[a]	2	0.075%	1	0.075
6: Determination of $N_{D,w,Q}$ at the 10 MV photon beam[f]	Normal	±0.3%[e]	1	0.3%	1	0.3
Combined standard uncertainty	Normal					0.741
Expanded uncertainty (k = 2)						1.5

[a]Derived from the variation limit or standard deviation in the realistic estimate of Table 1.

[b]Combining components 1-2-1, 1-2-2, and 1-2-3.

[c]Combining components 2-1 and 2-2.

[d]Calibrated directly in MV photon beams.

[e]Quoted from Ref. [45].

[f]Uncertainty in the determination of a calibration coefficient of absorbed dose to water ($N_{D,w,Q}$) at a user beam quality Q using a polynomial approximation [45].

PDF, probability density function; SCD, source-chamber distance; M_{raw}, mean chamber reading; MU, monitor units; M_+ and M_-, mean-chamber readings obtained at polarizing voltages of -300 and $+300$ V, respectively; M_1 and M_2, mean-collected charges obtained at polarizing voltages of $+300$ and $+100$ V, respectively; $N_{D,w,Q}$, calibration coefficient of absorbed dose to water at a user beam quality Q

Supplementary Table 54. Conservative uncertainty in the reference dosimetry of the 4 MV photon beam using the ionization chamber calibrated in MV photon beams.

Component of uncertainty	PDF		Divisor	Standard uncertainty	Sensitivity coefficient	Uncertainty contribution (%)
	Shape	Variation limit/ Standard deviation				
1: Reference conditions						
1-1: SCD setting	Uniform	±2 mm[a]	1.73	1.2 mm	0.2%/mm	0.23
1-2: Chamber setting	Normal	±0.5 mm[a]	1	0.5 mm	0.66%/mm	0.33
1-3: Field-size setting	Uniform	±2 mm[a]	1.73	1.2 mm	0.20%/mm	0.23
2: Charge measurement	Normal	±0.6%[a]	1	0.6%	1	0.6
3: Long-term stability of ion chamber	Normal	±0.3%[a]	1	0.3%	1	0.3
4: Correction for influence quantities						
4-1: Pressure and temperature						
4-1-1: Instrument error in thermometer	Normal	±0.3 ℃[a]	1	0.3 ℃	0.34%/℃	0.10
4-1-2: Instrument error in barometer	Normal	±0.1 kPa[a]	1	0.1 kPa	0.99%/kPa	0.10
4-2: Polarity effect	Normal	±0.05%[a]	1	0.05%	1	0.05
4-3: Ion Recombination	Normal	±0.10%[a]	1	0.10%	1	0.10
4-4: Humidity	Normal	±0.15%[a]	1	0.15%	1	0.15
5: Calibration of dosimeter						
5-1: Ion chamber[b]	Normal	±0.5%[c]	1	0.5%	1	0.5
5-2: Electrometer	Normal	±0.09%[a]	1	0.09%	1	0.09
6: Determination of $N_{D,w,Q}$ for the 4 MV photon beam[d]	Normal	±0.3%[c]	1	0.3%	1	0.3
Combined standard uncertainty	Normal					1.03
Expanded uncertainty (k = 2)						2.1

[a]Derived from the variation limit or standard deviation in the conservative estimate of Table 1.

[b]Calibrated directly in MV photon beams.

[c]Quoted from Ref. [45].

[d]Uncertainty in the determination of a calibration coefficient of absorbed dose to water ($N_{D,w,Q}$) at a user beam quality Q using a polynomial approximation [45].

PDF, probability density function; SCD, source-chamber distance; $N_{D,w,Q}$, calibration coefficient of absorbed dose to water at a user beam quality Q

Supplementary Table 55. Conservative uncertainty in the reference dosimetry of the 6 MV photon beam using the ionization chamber calibrated in MV photon beams.

Component of uncertainty	PDF		Divisor	Standard uncertainty	Sensitivity coefficient	Uncertainty contribution (%)
	Shape	Variation limit/ Standard deviation				
1: Reference conditions						
1-1: SCD setting	Uniform	±2 mm[a]	1.73	1.2 mm	0.2%/mm	0.23
1-2: Chamber setting	Normal	±0.5 mm[a]	1	0.5 mm	0.57%/mm	0.29
1-3: Field-size setting	Uniform	±2 mm[a]	1.73	1.2 mm	0.15%/mm	0.17
2: Charge measurement	Normal	±0.6%[a]	1	0.6%	1	0.6
3: Long-term stability of ion chamber	Normal	±0.3%[a]	1	0.3%	1	0.3
4: Correction for influence quantities						
4-1: Pressure and temperature						
4-1-1: Instrument error in thermometer	Normal	±0.3 ℃[a]	1	0.3 ℃	0.34%/℃	0.10
4-1-2: Instrument error in barometer	Normal	±0.1 kPa[a]	1	0.1 kPa	0.99%/kPa	0.10
4-2: Polarity effect	Normal	±0.05%[a]	1	0.05%	1	0.05
4-3: Ion Recombination	Normal	±0.10%[a]	1	0.10%	1	0.10
4-4: Humidity	Normal	±0.15%[a]	1	0.15%	1	0.15
5: Calibration of dosimeter						
5-1: Ion chamber[b]	Normal	±0.5%[c]	1	0.5%	1	0.5
5-2: Electrometer	Normal	±0.09%[a]	1	0.09%	1	0.09
6: Determination of $N_{\mathrm{D,w,Q}}$ for the 6 MV photon beam[d]	Normal	±0.3%[c]	1	0.3%	1	0.3
Combined standard uncertainty	Normal					1.01
Expanded uncertainty (k = 2)						2.1

[a]Derived from the variation limit or standard deviation in the conservative estimate of Table 1.

[b]Calibrated directly in MV photon beams.

[c]Quoted from Ref. [45].

[d]Uncertainty in the determination of a calibration coefficient of absorbed dose to water ($N_{\mathrm{D,w,Q}}$) at a user beam quality Q using a polynomial approximation [45].

PDF, probability density function; SCD, source-chamber distance; $N_{\mathrm{D,w,Q}}$, calibration coefficient of absorbed dose to water at a user beam quality Q

Supplementary Table 56. Conservative uncertainty in the reference dosimetry of the 10 MV photon beam using the ionization chamber calibrated in MV photon beams.

Component of uncertainty	PDF		Divisor	Standard uncertainty	Sensitivity coefficient	Uncertainty contribution (%)
	Shape	Variation limit/ Standard deviation				
1: Reference conditions						
1-1: SCD setting	Uniform	±2 mm[a]	1.73	1.2 mm	0.2%/mm	0.23
1-2: Chamber setting	Normal	±0.5 mm[a]	1	0.5 mm	0.47%/mm	0.24
1-3: Field-size setting	Uniform	±2 mm[a]	1.73	1.2 mm	0.11%/mm	0.13
2: Charge measurement	Normal	±0.6%[a]	1	0.6%	1	0.6
3: Long-term stability of ion chamber	Normal	±0.3%[a]	1	0.3%	1	0.3
4: Correction for influence quantities						
4-1: Pressure and temperature						
4-1-1: Instrument error in thermometer	Normal	±0.3 ℃[a]	1	0.3 ℃	0.34%/℃	0.10
4-1-2: Instrument error in barometer	Normal	±0.1 kPa[a]	1	0.1 kPa	0.99%/kPa	0.10
4-2: Polarity effect	Normal	±0.05%[a]	1	0.05%	1	0.05
4-3: Ion Recombination	Normal	±0.1%[a]	1	0.10%	1	0.10
4-4: Humidity	Normal	±0.15%[a]	1	0.15%	1	0.15
5: Calibration of dosimeter						
5-1: Ion chamber[b]	Normal	±0.5%[c]	1	0.5%	1	0.5
5-2: Electrometer	Normal	±0.09%[a]	1	0.09%	1	0.09
6: Determination of $N_{D,w,Q}$ for the 10 MV photon beam[d]	Normal	±0.3%[c]	1	0.3%	1	0.3
Combined standard uncertainty	Normal					0.990
Expanded uncertainty (k = 2)						2.0

[a]Derived from the variation limit or standard deviation in the conservative estimate of Table 1.

[b]Calibrated directly in MV photon beams.

[c]Quoted from Ref. [45].

[d]Uncertainty in the determination of a calibration coefficient of absorbed dose to water ($N_{D,w,Q}$) at a user beam quality Q using a polynomial approximation [45].

PDF, probability density function; SCD, source-chamber distance; $N_{D,w,Q}$, calibration coefficient of absorbed dose to water at a user beam quality Q
